# Comprehensive profiling of extracellular vesicles in uveitis and scleritis enables biomarker discovery and mechanism exploration

**DOI:** 10.1186/s12967-023-04228-x

**Published:** 2023-06-15

**Authors:** Lingzi Wu, Lei Zhou, Jinying An, Xianfeng Shao, Hui Zhang, Chunxi Wang, Guixia Zhao, Shuang Chen, Xuexue Cui, Xinyi Zhang, Fuhua Yang, Xiaorong Li, Xiaomin Zhang

**Affiliations:** 1 Tianjin Key Laboratory of Retinal Functions and Diseases, Tianjin Branch of National Clinical Research Center for Ocular Disease, Eye Institute and School of Optometry, Tianjin Medical University Eye Hospital, Tianjin, 300384 China; 2 Department of Applied Biology and Chemical Technology, School of Optometry, Research Centre for SHARP Vision (RCSV), The Hong Kong Polytechnic University, Hong Kong, China; 3 Centre for Eye and Vision Research (CEVR), 17W Hong Kong Science Park, Hong Kong, China; 4 State Key Laboratory of Proteomics, National Center for Protein Sciences (Beijing), Beijing Proteome Research Center, Beijing Institute of Lifeomics, Beijing, China; 5 Tangshan Eye Hospital, Tangshan, China

**Keywords:** Plasma, Small extracellular vesicles, Large extracellular vesicles, Uveitis, Scleritis, Proteomic profiles, Biomarkers, Connectivity map

## Abstract

**Background:**

Uveitis and posterior scleritis are sight-threatening diseases with undefined pathogenesis and accurate diagnosis remains challenging.

**Methods:**

Two plasma-derived extracellular vesicle (EV) subpopulations, small and large EVs, obtained from patients with ankylosing spondylitis-related uveitis, Behcet's disease uveitis, Vogt-Koyanagi-Harada syndrome, and posterior scleritis were subjected to proteomics analysis alongside plasma using SWATH-MS. A comprehensive bioinformatics analysis was performed on the proteomic profiles of sEVs, lEVs, and plasma. Candidate biomarkers were validated in a new cohort using ELISA. Pearson correlation analysis was performed to analyze the relationship between clinical parameters and proteomic data. Connectivity map database was used to predict therapeutic agents.

**Results:**

In total, 3,668 proteins were identified and over 3000 proteins were quantified from 278 samples. When comparing diseased group to healthy control, the proteomic profiles of the two EV subgroups were more correlated with disease than plasma. Comprehensive bioinformatics analysis highlighted potential pathogenic mechanisms for these diseases. Potential biomarker panels for four diseases were identified and validated. We found a negative correlation between plasma endothelin-converting enzyme 1 level and mean retinal thickness. Potential therapeutic drugs were proposed, and their targets were identified.

**Conclusions:**

This study provides a proteomic landscape of plasma and EVs involved in ankylosing spondylitis-related uveitis, Behcet's disease uveitis, Vogt-Koyanagi-Harada syndrome, and posterior scleritis, offers insights into disease pathogenesis, identifies valuable biomarker candidates, and proposes promising therapeutic agents.

**Supplementary Information:**

The online version contains supplementary material available at 10.1186/s12967-023-04228-x.

## Introduction

Uveitis and scleritis are common sight-threatening diseases characterized by intraocular inflammation involving the uvea, retina, sclera, and adjacent structures. These are collective terms for a variety of intraocular inflammations, including ankylosing spondylitis (AS)-related anterior uveitis (AU), Behcet's disease (BD) uveitis, posterior scleritis (pSCL), and Vogt-Koyanagi-Harada syndrome (VKH), etc. Visual impairment due to uveitis accounts for 5–10% worldwide [1]. AS-related AU is the most common extra-articular manifestation of AS, accounting for 26.3% of all AU in China [2, 3]. It has an acute onset, severe inflammation, and a tendency for recurrence [4]. BD uveitis and VKH are two types of the most common panuveitis entities in China, accounting for 16.5% and 15.9% respectively [5]. 45–90% of patients with confirmed BD have ocular involvement, with the rate of blindness being high within 6–10 years without aggressive treatment [6]. VKH is a multisystem, multiphasic immune-mediated disorder with its most dire manifestations affecting the eyes, which can lead to blindness. pSCL accounts for 2–12% of all scleritis cases and usually causes significant periocular pain or headache, as well as vision loss [7]. Among all types of scleritis, decreased visual acuity is most common (60%) in pSCL [8]. Currently, the diagnosis of most types of uveitis and scleritis is based on clinical symptoms without laboratory confirmation. Glucocorticoids, immunosuppressants, and biological agents are the primary drugs prescribed for ocular immune diseases, however, individuals with different types of diseases may respond differently to various therapeutic protocols. Therefore, understanding the underlying pathogeneses of these diseases is important. Moreover, the identification of highly sensitive biomarkers is essential for improving clinical decision-making and clinical outcomes as well as avoiding unnecessary treatment.

The International Society for Extracellular Vesicles (ISEV) defines “extracellular vesicles” (EVs) as the generic term for membranous vesicles naturally released by cells [9]. They are enclosed by a lipid bilayer and unable to replicate. As mediators of cell communication, EVs generally reflect the function of the original cells and their physiological state [10, 11]. EVs are secreted by almost all cell types, including immune cells, endothelial cells, etc. [12]. The content of EVs (i.e., DNA, RNA, protein, and lipid) is protected from proteases and nucleases by its phospholipid bilayer. EVs can regulate cell proliferation, migration, immune response, and angiogenesis [13]. Moreover, they were found to be involved in regulating the physiological and pathological processes of numerous diseases, including neurodegenerative diseases, infections, cancer, and autoimmune diseases [11].

In recent years, the EV-mediated pathophysiology and its application as a source of novel biomarkers and potential therapeutic agents have received increasing interest. They can transmit viruses, deliver inflammatory signals, and facilitate neurological regeneration, neovascularization, tumor progression, and metastasis [14]. EVs are associated with ocular disease progression. One previous study showed that EVs are useful for detecting, monitoring, and predicting retinal diseases and optic nerve damage [15]. Some EVs, especially those with smaller diameters, such as exosomes, can easily cross biological barriers, including the blood–ocular barrier, allowing EV-mediated communication among different tissues and organs [16]. The EVs from diseased tissues will increase in a pathological state [17], and the changes in the component of circulating EVs can reflect the state of the disease. Therefore, proteomic analysis of circulating EVs might help reveal the pathogenesis of ocular immune diseases and identify useful biomarkers. Currently, most existing research on the pathogenesis of uveitis is based on uveitis animal models, which have limitations in simulating complex and heterogeneous human diseases. Therefore, studies using human samples could provide more information about specific types of uveitis or scleritis.

As suggested by the ISEV, EVs can be differentiated into “small EVs” (sEVs; e.g., exosomes) and “large EVs” (lEVs; e.g., microvesicles) according to their size. Here, we performed proteomic analysis of plasma and plasma-derived sEVs and lEVs obtained from patients with AS-related-AU, BD uveitis, pSCL, and VKH. We identified disease-specific biomarker panels for four diseases and further explored the intrinsic pathological processes associated with these diseases using global proteomics analyses of EVs and plasma, providing a molecular basis for future studies. We also performed the correlation analysis between clinical parameters and protein quantitative value to explore the molecular mechanisms of clinical alterations. Finally, we offered novel pharmaceutical options for disease treatment based on the proteome changes of different diseases. Collectively, our study presents the proteomic landscape of plasma and EVs from patients with several immune ocular disease subtypes, providing information for in-depth disease understanding, the development of hematological diagnostic tools, and the improvement of therapeutic strategies. Notably, AS-related-AU, BD uveitis and pSCL are abbreviated as AS, BD and SCL in the following sections.

## Material and methods

### Patient information and ethical approval

Plasma samples were collected from patients who visited the Tianjin Medical University Eye Hospital and Tangshan Eye Hospital between November 2017 and March 2022. Patients with a confirmed diagnosis of AS (HLA-B27 positive), BD, VKH, and SCL were recruited in the discovery phase of this study and grouped (AS, BD, SCL, and VKH, n = 16). Individuals with diabetes, HBV infectors or carriers, thyroid disease, other systemic immune diseases, metabolic diseases, and cataracts with a history of uveitis were excluded.

The plasma samples were obtained while the ocular inflammation was at the acute stage for patients with AS and VKH, and the active phase for patients with BD and SCL. Standardization of uveitis nomenclature working group characterized the acute phase as sudden onset and limited duration [18]. Accordingly, the acute phase of AS was defined as a sudden onset (less than 7 days). The acute phase of VKH was defined as the first attack of short duration with bilateral diffuse choroiditis and bullous serous retinal detachment [19]. For patients with BD, the grade of anterior chamber cells or vitreous opacity was ≥ 2 + ; for patients with SCL, the B-scan showed apparent thickening of the eyeball wall. For the control group, healthy volunteers and patients with age-related cataracts of matched ages and sexes were selected. Notably, patients enrolled in the AS and VKH groups had never systemically used glucocorticoids or immunosuppressants.

All the procedures complied with the World Medical Association Declaration of Helsinki. Ethical approval was obtained from the Ethics Committee of the Tianjin Medical University Eye Hospital, Tianjin, China (approval number. 2019KY(L)-34). All participants provided written informed consent.

### Sample collection and EV isolation

Blood samples were centrifuged at 1,800 × g for 15 min at 4 °C. The subsequent centrifugation procedures were operated at 4 °C. The supernatant was collected and stored at − 80 °C until further processing. To isolate EVs, 2 mL plasma was thawed on ice and centrifuged at 2,000 × g for 15 min to remove cell debris and other impurities. The supernatant was diluted with PBS (Gibco, USA) and ultracentrifuged at 80,000 × g for 30 min (rotor, SW 41 Ti, Beckman Coulter, USA). The pellet was resuspended in PBS and centrifuged at 15,000 × g for 30 min. The resulting pellet containing lEVs was resuspended in 200 μL 8 M urea lysis buffer (Sigma-Aldrich, Germany) containing protease inhibitor (Roche, Germany) for subsequent use. The resulting supernatant was ultracentrifuged at 110,000 × g for 2 h. The pellet was resuspended in PBS and passed through a 0.22 µm filter (Merck, Germany), and ultracentrifuged at 150,000 × *g* for 2 h. The resulting pellet containing enriched sEVs was resuspended in 300 μL 8 M urea lysis buffer for further processing.

### Nanoparticle tracking analysis

Freshly isolated EVs samples were resuspended with PBS and measured using a NanoSight NS300 instrument (NanoSight, UK) with a 488 nm laser and a sCMOS camera. Three 60 s videos were captured for each sample at 25 °C. For analysis, NTA 3.3 software (Dev Build 3.3.104) was used and the detection threshold was set to 5.

### Western blot analysis and coomassie brilliant blue staining

The obtained sEVs and lEVs from plasma were resuspended in 0.1% (v/v) Triton lysis buffer (Solarbio, China), and the sample loading amount normalized using bicinchoninic acid protein quantification (Solarbio). Proteins (10 μg) were separated using 10% sodium SDS-PAGE. The separated proteins were transferred onto PVDF membranes blocked with 5% (w/v) non-fat dry milk and incubated at room temperature (RT) for 1 h. The membranes were washed and incubated overnight at 4 °C with primary antibodies against CD63 (1:1000, ab92726, Abcam, UK), HSP70 (1:1000, 66,183-1-IgM, Proteintech, China), Apo-A1 (1:5000, 14,427-1-AP, Proteintech), and Grp94 (1:2000, 60,012-2-Ig, Proteintech). The following day, the membranes were washed and incubated at RT for 1 h with secondary antibodies (goat anti-rabbit/mouse IgG H&L chain conjugated to HRP, 1:2000, Abcam). All washes comprised 10 min washes with 0.05% (v/v) Tween-20 (Solarbio) at least three times. The antigen–antibody binding complexes were detected using an enhanced chemiluminescence reagent (Thermo, USA), and the bands were visualized using a chemiluminescence imager (Tanon, China).

For coomassie brilliant blue (CBB) staining, the proteins (10 μg) were separated as before using SDS-PAGE, and the resulting gels were immersed in the Coomassie dye solution (BeyoBlue, China) for 2 h at RT and eluted overnight with ultrapure water.

### Transmission electron microscopy

Transmission electron microscopy (TEM) was used to directly observe the morphology of the EVs. Freshly isolated EVs samples were resuspended with PBS and subsequently placed on a formvar copper grid until they were absorbed, stained with 1% (w/v) uranyl acetate, and air-dried. The grids were viewed under an electron microscope (HITACHI, HT7700, Japan).

### Sample preparation for sequential window acquisition of all theoretical fragment ions-mass spectrometry acquisition

Samples of sEVs, lEVs, and plasma were lysed using an 8 M urea lysis buffer. After reduction and alkylation, the sample was loaded onto a Vivacon 500 centrifugal concentrator (Sartorius, Germany) and digested with trypsin (Promega, USA) at a 50:1 protein-to-enzyme ratio for 16 h at 37 °C. The resulting tryptic peptides were eluted with 50 mM NH_4_HCO_3_ (BioUltra, > 99.5%, Sigma-Aldrich), and digestion was terminated with 1% (v/v) FA (LiChroPUR, 98–100%, Merck). The peptides were dried using an integrated SpeedVac (Thermo).

### Generation of the spectral library: information-dependent acquisition

A reference spectral library was built for sequential window acquisition of all theoretical fragment ions-mass spectrometry (SWATH-MS) acquisition. To construct a cohort-specific spectral library, an aliquot from each study sample including sEVs and lEVs was pooled. To further enhance the depth of the spectral library, sEVs, lEVs, and high-abundance depleted plasma samples from another 12 healthy individuals were pooled together with the above study sample pool followed by fractionation and LC–MS/MS analysis in information-dependent acquisition (IDA) mode. The plasma samples for library construction were subjected to high-abundance protein depletion with the Pierce Top 12 Abundant Protein Depletion Spin Columns (85,165, Thermo). These samples were fractionated into nine fractions with a high pH reversed-phase micro-column Durashell C18 (DC930010-L, Agela, China). Each fraction was analyzed using a nano-LC 415 (Eksigent Technologies, USA) coupled with an electrospray ionization Triple TOF 6600 mass spectrometers (AB Sciex, USA). The peptides were loaded onto a homemade trap column (100 µm × 2 cm, Durashell C18 3 μm, 120 Å) and separated on a homemade analytical column (150 µm × 15 cm, Durashell C18 1.9 μm, 120 Å). The flow rates of the trap column for loading and the analytical column for elution were 3 and 0.5 μL/min, respectively. The peptides were resolved with 120 min nano-LC gradient (linear gradient from 5 to 80% HPLC buffer B [0.1% formic acid in acetonitrile] in 105 min and then to 5% buffer B in 15 min). For data acquisition in traditional IDA mode, TOF–MS spectra were recorded in a mass range of 300–1500 (m/z) with 0.25 s of an accumulation time. The criteria for ion selection included a charge state between + 2 and + 5, a mass tolerance of 50 ppm, and an intensity above 150 counts. Tandem MS in the mass range of 100–1500 m/z was performed using dynamic fragmentation collision energy and high-sensitivity scanning mode with 0.04 s of an accumulation time.

### Extraction of quantitative values-data independent acquisition

For SWATH-MS, the same chromatographic parameters described above were used in the IDA model for ion library construction. The accumulation time of TOF–MS was set at 0.05 s, and the high-sensitivity scanning mode was used for MS/MS. Furthermore, 100 variable windows with 30 ms accumulation time were used based on the SWATH variable window calculator V1.1. The scanning mass range was 300–1500 Da. Typically, 1 µg, 0.5 µg, or 1 µg of peptides for sEVs, lEVs, and plasma were injected for analysis, respectively.

### Enzyme linked immunosorbent assay

Quantification of mannan-binding lectin serine protease 1 (MASP1, Cloud-Clone Corp, China), activated leukocyte cell adhesion molecule (ALCAM, Cusabio, China), metalloproteinase inhibitor 3 (TIMP3, Cusabio), receptor-type tyrosine-protein phosphatase eta (PTPRJ, FineTest, China), follistatin-related protein 1 (FSTL1, FineTest), tripartite motif 21 (TRIM21, FineTest), TANK-binding kinase 1 (TBK1, FineTest), mannose-binding protein C (MBL2, FineTest), and endothelin-converting enzyme 1 (ECE1, FineTest) was performed using commercial ELISA kits according to the manufacturer’s instructions. The sEVs and lEVs samples were resuspended in 150 μl and 120 μl of 0.1% Triton lysis buffer, respectively. The incubation steps in ELISA were performed at 37 °C. Briefly, 100 μL of diluted plasma or EV sample was incubated with the respective capture antibody for 90 min, followed by the detection antibody for 60 min. HRP-conjugated secondary antibodies were added after washing and incubated for 30 min. Tetramethylbenzidine solution was added after washing and incubated for 10–20 min. The reaction was terminated using a stop solution provided by the kits. The OD value at 450 nm referenced to 540 nm was measured using a Tecan Infinite 200Pro plate reader (Zurich, Switzerland). The concentrations were determined from a standard curve generated using the standards provided in the kit.

### Prediction of potential therapeutic agents using connectivity map

Potential candidate drugs were obtained based on differentially expressed proteins (DEPs) using Connectivity Map (CMap, https://clue.io/) [20]. CMap with the L1000 platform, a low-cost and high-throughput reduced representation expression profiling method, was used to query potential drugs based on upregulated and downregulated signature proteins [21]. The false discovery rate (FDR) was set to 0.05 when filtering the result of CMap. The negative connectivity score, which indicates the inhibition effect, was obtained according to its pattern-matching algorithms. The background and indication of drugs were obtained from DrugBank (https://go.drugbank.com/), PubChem (https://pubchem.ncbi.nlm.nih.gov/), and ChEBI (https://www.ebi.ac.uk/chebi/). The drug–protein interaction network was constructed using the Comparative Toxicogenomics Database (CTD, http://ctd.mdibl.org), a robust and publicly available database that provides information on chemical–gene–disease relationships from the literature [22].

### Proteomic profiling analysis and visualization

The original IDA spectral data were analyzed by the ProteinPilot software (v. 5.0.1; AB Sciex). The UniProt human SwissProt database (released July 2019) containing 20,431 annotated proteins with an FDR < 1% was used for protein identification and library generation. Data-independent acquisition results were processed using the SWATH Acquisition Micro app (v. 2.0) in the PeakView (v. 2.2; AB Sciex) software according to the spectral library constructed as described above. This process used an extraction window of 12 min and the following conditions: six peptides and, six transitions, excluding shared and modified peptides; an XIC width of 50 ppm; peptide confidence > 99%; and FDR < 1%. An iRT (indexed retention time; Ki-3002–02, BIOGNOSYS, Switzerland) was used to calibrate the retention time. The peak areas were exported as quantitative values.

Raw mass spectrometry data were log2-transformed and quantile normalized using the “normalize” function in the “preprocessCore” package in R. Differential analysis was conducted using the “t.test” function in the “stats” package in R. Proteins with a fold change (FC) > 1.5 or < − 1.5 and p-value < 0.05 were considered DEPs. Pearson correlation coefficients and p-values were computed using the “rcorr” function in R. Heatmap, violin plots, bubble plots, principal component analysis (PCA) score plots, chord diagrams, and boxplots were visualized using the “pheatmap”, “ggplot”, “prcomp”, and “circlize” packages in R, respectively. Gene set variation analysis (GSVA) and gene set enrichment analysis (GSEA) were performed using the “GSVA” and “clusterProfiler” packages in R, respectively. GSVA was applied using the Gene Ontology (GO) biological processes gene sets (c2.cp.v7.1.symbols.gmt). GSEA was applied using two databases, GO biological processes (c5.go.bp.v7.5.1.symbols.gmt) and Reactome gene sets (c2.cp.reactome.v7.5.1.symbols.gmt). Partial least squares-discriminant analysis (PLS-DA) was implemented with the “mixOmics” package in R. Venn diagrams, scatter plots, rose charts, balloon plots, bar plots, polar bar plots, stack bar plot, volcano plots, receiver operating characteristic curves (ROC), and rank plots were plotted using the online biomedical visualization software Hiplot (https://hiplot.com.cn) and an online platform (https://bioinformatics.com.cn) and adjusted using Adobe Illustrator (Adobe, USA). GO and Kyoto Encyclopedia of Genes and Genomes (KEGG) pathway enrichment analyses were performed using the Metascape online analysis software (http://metascape.org) [23]. Images of endoplasmic reticulum (ER), mitochondrion and ocular were generated by SMART PPT (https://smart.servier.com). The hub proteins were identified using the plug-in cytoHubba in Cytoscape (http://apps.cytoscape.org/apps/cytohubba).

### Statistics analysis

Statistical analyses were performed using the SPSS software (v. 24.0; IBM Corp, USA). Normal data are presented as the mean ± standard deviation, and non-normal data as the median ± interquartile ranges. For normal data with even variance, Student’s t-tests were used for analysis, otherwise, Mann–Whitney rank tests were used. A comparison of sex between the two groups was performed using the chi-square test. Significant differences are indicated with asterisks as follows: *p < 0.05, **p < 0.01, ***p < 0.001, and ****p < 0.0001. The predicted probability of combined ROC estimation was carried out using bivariate logistic regression analysis. ROC curve analysis was performed by MedCalc (v. 19.6.4; MedCalc Software, Belgium) to assess the sensitivity, specificity, predictive value, likelihood ratio, and odds ratio.

## Results

### Morphological and protein composition characterization of plasma-derived EVs

The differential ultracentrifugation-based technique, which is considered a “gold standard” approach for EV separation [24], was used to separate plasma-derived sEVs and lEVs (Additional file 1: Fig. S1A). Nanoparticle tracking analysis (NTA) demonstrated a size distribution with mean diameters for sEVs-enriched samples and lEVs-enriched samples were 107.2 ± 43.0 nm and 214.2 ± 76.3 nm respectively (Additional file 1: Fig. S1B). In the sEV fraction, 94.4% of the EVs had a range of 50–200 nm, whereas 97.0% of the EVs had a range of 100–400 nm in the lEV fractions. TEM revealed that sEVs had a classic cup-shaped structure with a diameter of approximately 100 nm, whereas lEVs were round-shaped vesicles with a diameter of approximately 400 nm (Additional file 1: Fig. S1C).

According to the recommendations of Minimal information for studies of extracellular vesicles (2018) [9], four categories of EV-related proteins must be analyzed to verify the presence of EVs and assess the purity of the EV-enriched samples. CD63 and HSP70, specific protein markers, were expressed in all EVs, whereas the negative purity control marker Apo-A1 was expressed poorly in the EVs (Additional file 1: Fig. S1D). The absence of Grp94, an ER marker, demonstrated the reliability of sEVs isolation. Conversely, Grp94 was detected in lEVs. The CBB-stained gel revealed different protein profiles among the sEVs, lEVs, and plasma samples (Additional file 1: Fig. S1E). These results demonstrate that the isolated EVs in this study meet the criteria for subsequent experiments.

### Proteomic analysis reveals specific proteomic profiles in EVs and plasma

**Fig. 1:**
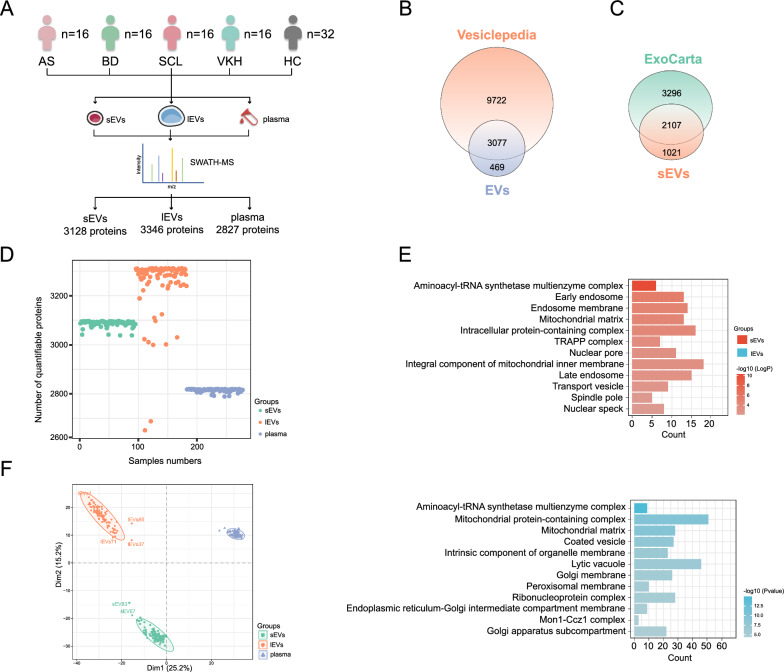
Proteomic analysis reveals specific proteomic profiles in EVs and plasma. **A** Workflow for acquiring proteomic data. **B** Venn diagram showing the overlap between the EVs protein database from Vesiclepedia and the identified proteins in our EVs samples. **C** Venn diagram showing the overlap between the exosomal proteins from the ExoCarta databases and the identified proteins in our sEVs samples. **D** Scatter plot displays the distribution of the number of quantifiable proteins detected per sample. **E** Bar plots showing GO terms in the cellular component category enriched by proteins specifically identified in sEVs and lEVs. **F** Principal component analysis score plots based on the 2,387 shared proteins in the three components. *AS* ankylosing spondylitis-related acute anterior uveitis, *BD* Behcet's disease uveitis, *SCL* posterior scleritis, *VKH* Vogt-Koyanagi-Harada syndrome, *HC* healthy control, *SWATH-MS* sequential window acquisition of all theoretical fragment ions-mass spectrometry

The schematic workflow for obtaining proteomic data is illustrated in Fig. 1A. In the discovery phase, 288 biological samples from 96 enrolled participants were separately used for the SWATH assay of sEVs, lEVs, and plasma, and 278 biological samples were finally analyzed as 10 samples did not meet the loading requirements due to the low yield. Additional file 6: Table S1 lists the demographic and baseline characteristics of the participants in the discovery cohorts.

To obtain a larger spectral library, IDA data from sEVs, lEVs, and plasma were combined to establish a library of 3,978 proteins. Our proteomics data identified 3,668 proteins, with 3,128, 3,346, and 2,827 proteins in sEVs, lEVs, and plasma, respectively, at an FDR < 1% across all samples using SWATH-MS acquisition (Additional file 6: Table S1). The median coefficient of variation [25, 26] values for protein quantification in sEVs/lEVs/plasma samples were 16.42%, 16.73%, and 12.58%, respectively. The percentages of the coefficient of variation < 20% were 83.38%, 87.06%, and 98.23%, respectively (Additional file 1: Fig. S1F). This evidence provided support for the further application of the sEVs/lEVs/plasma SWATH-MS workflow.

Nearly 90% of the identified proteins in our EVs samples overlapped with the EVs protein database from Vesiclepedia (http://microvesicles.org), which is a manually curated compendium of molecular data (lipid, RNA, and protein) identified in various types of EVs [27] (Fig. 1B, Additional file 7: Table S2). In addition, of the 3,128 proteins identified in our sEVs samples, nearly 70% overlapped with exosomal proteins included in the ExoCarta database (http://exocarta.org) (Fig. 1C, Additional file 7: Table S2). Proteins with more than one-third of the missing values in all samples were excluded. The total number of quantifiable proteins from the SWATH-MS analysis was 3,658, with 3,097, 3,312, and 2,820 for sEVs, lEVs, and plasma, respectively (Additional file 6: Table S1). The median and mode of quantifiable protein numbers (sEVs, lEVs, and plasma samples: 95, 87, and 96, respectively) were 3,091 and 3,093 in sEVs (ranging from 3,038–3,097), 3,294 and 3,312 in lEVs (ranging from 2651–3312), and 2,817 and 2,818 in plasma (ranging from 2789–2820) (Fig. 1D).

GO enrichment analysis of the cellular component category revealed that 211 proteins specifically identified in sEVs were strongly associated with endosomes (Fig. 1E, Additional file 1: Fig. S1G, Additional file 7: Table S2). Conversely, 426 proteins uniquely identified in lEVs were related to the Golgi apparatus and ER (Fig. 1E). This difference between sEVs and lEVs is attributed to the distinct secretion process of exosomes and microvesicles. Exosomes are produced through the formation of early and late endosomes, whereas, microvesicles are mostly formed with the involvement of intracellular compartments including the peroxisomes, Golgi apparatus, and ER. PCA based on the expression value of 2,387 shared proteins showed that the two EV subpopulations and plasma were separated and far apart from each other, indicating that their proteomic profiles were markedly different (Fig. 1F, Additional file 1: Fig. S1H). These results present an overview of the proteomic data in our study and demonstrate the specific proteomic profile of sEVs, lEVs, and plasma.

### Differential proteomic signature of the four ocular immune diseases

**Fig. 2:**
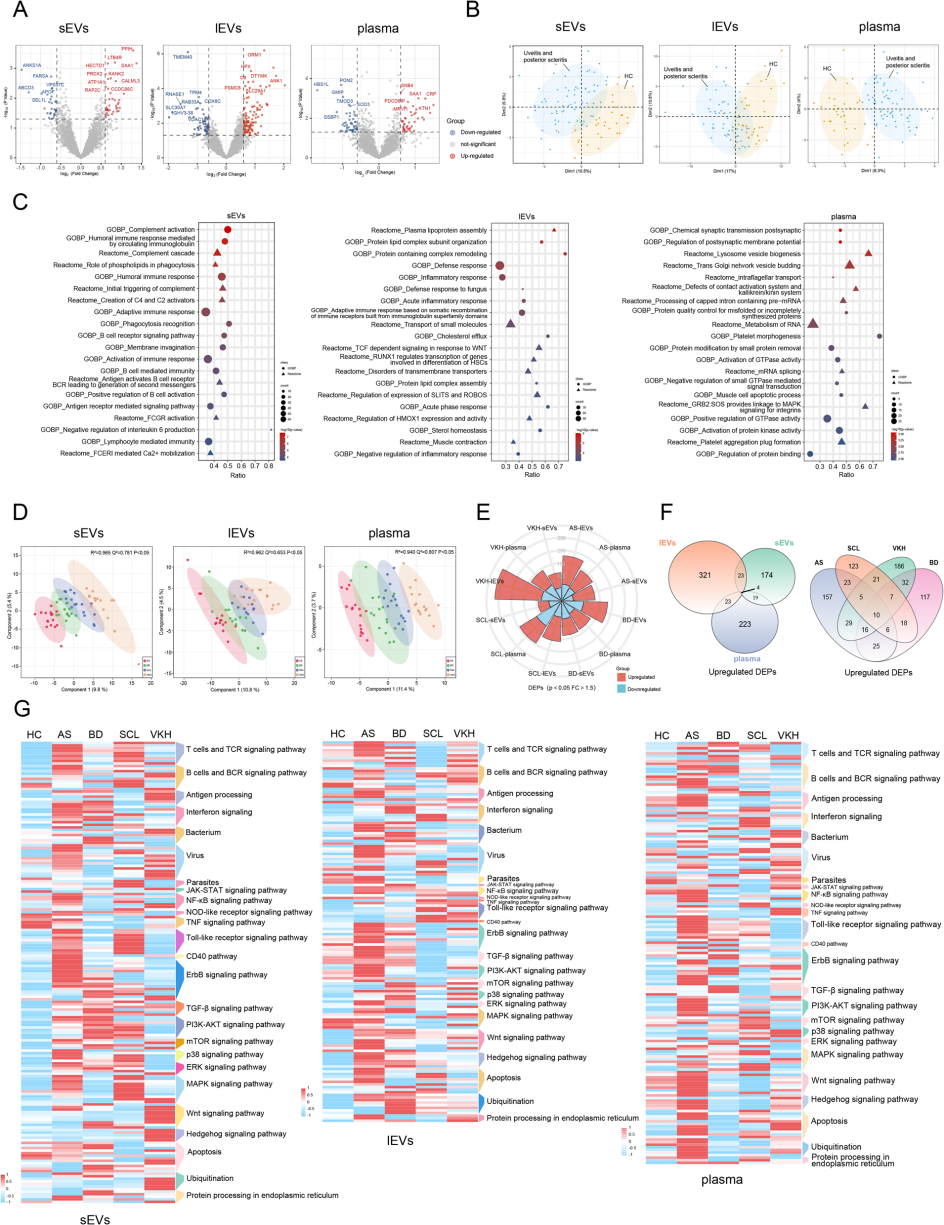
Differential proteomic signature of the four ocular immune diseases.** A** Volcano plots indicating DEPs between diseased patients and HCs. **B** Principal component analysis results showing the expression patterns of HCs and diseased patients based on the DEPs between diseased patients and HCs. **C** Bubble plots describing the GSEA results of sEVs, lEVs, and plasma. **D** Score plots showing the PLS-DA model using proteins in sEVs (R^2^ = 0.965, Q^2^ = 0.781, p-value < 0.05, 3 components), lEVs (R^2^ = 0.962, Q^2^ = 0.653, p-value < 0.05, 4 components) and plasma (R^2^ = 0.940, Q^2^ = 0.807, p-value < 0.05, 2 components); The number of PLS-DA components was determined according to the highest Q^2^ value. **E** Rose-Chart showing the DEPs in the individual components of the four diseases. **F** Venn diagrams showing the overlap of significantly upregulated DEPs in the four diseases among sEVs, lEVs, and plasma, and the overlap of significantly upregulated DEPs in the three components among the four diseases. **G** Heatmaps displaying the score of canonical intracellular pathways by GSVA of the four diseases and HC. Red represents relative activation, while blue represents relative inhibition. *AS* ankylosing spondylitis-related acute anterior uveitis, *BD* Behcet's disease uveitis, *SCL* posterior scleritis, *VKH* Vogt-Koyanagi-Harada syndrome, *HC* healthy control, *DEP* differentially expressed protein

Patients with the four ocular immune diseases were merged as the diseased group, and the healthy participants served as normal controls. To illustrate the signature of diseased individuals, we analyzed the differences in the proteomic profiles of the EV and plasma between diseased and healthy control (HC) groups. A list of DEPs (|FC|> 1.5, p < 0.05, n = 76/177/92 for sEVs/lEVs/plasma) that could serve as potential protein biomarkers for identifying individuals at high risk of uveitis and SCL was identified (Fig. 2A, Additional file 8: Table S3). Several inflammation-inducible acute-phase proteins ranked at the top, such as serum amyloid A-1 protein (SAA1), orosomucoid-1 (ORM1), complement component C9 (C9), and c-reactive protein (CRP). A PCA based on these DEPs (Additional file 8: Table S3) showed that the diseased individuals were clustered together and separated from the HCs (Fig. 2B).

In addition, we performed GSEA using the FC value between the diseased group and healthy control of all quantifiable proteins and listed the top 20 entries in EVs and plasma ranked by p-values (Fig. 2C, Additional file 8: Table S3). Entries enriched in EVs include complement activation, acute inflammatory responses, and adaptive immune responses, which are critical processes in autoimmune diseases. However, the GSEA results in the plasma were almost independent of the immune response.

The supervised PLS-DA score plot showed a clear separation of the four diseases with a 95% confidence interval using the top 200 proteins in sEVs according to the variable importance in projection (VIP) scores (Fig. 2D). Similar results were obtained for lEVs and plasma using the top 300 and 100 proteins, respectively. Additional file 1: Fig. S1I shows the top 20 critical proteins for separation ranked by their VIP scores in sEVs, lEVs, and plasma. To evaluate the robustness of the PLS-DA models and avoid overfitting, permutation tests (100 random permutations) and cross-validation analyses (tenfold cross-validation method) were performed. The three PLS-DA models were considered valid because the permutation test p-value was < 0.05 and the Q^2^ value was > 0.4. These results demonstrate notable differences among the four diseases both in EV and plasma samples.

To achieve a deep and comprehensive understanding of each disease, we conducted a comparative proteomic analysis of each of the four diseases with HCs. The number of DEPs (|FC|> 1.5, p < 0.05) for each set of the four diseases, including those upregulated and down-regulated, is shown in Fig. 2E (Additional file 8: Table S3). The overlap shown in the Venn diagram (Fig. 2F, Additional file 1: Fig. S1J) was < 1%, suggesting that DEPs related to the disease varied considerably among sEVs, lEVs, and plasma. Moreover, the overlap of DEPs species among the four diseases was minimal, indicating that the main intrinsic pathogenesis of ocular immune diseases was different.

To understand the four diseases on a global scale, GSVA was performed using the expression value of all quantifiable proteins and the results are presented in Fig. 2G (Additional file 8: Table S3). The heatmap presents the mean GSVA scores for HC, uveitis, and SCL in terms of canonical intracellular pathways. Figure 2G clearly shows that the changes in biological processes in the diseased group were different from those in the HC group. More importantly, this diagram showed the relative activation and inhibition states of classical pathways for each disease, proving the commonalities and differences among these four diseases. These results highlight the characteristic protein signature of diseased individuals and the significant differences among the four subtypes.

### Vesicle-associated proteomics reveals possible disease pathogenesis

**Fig. 3:**
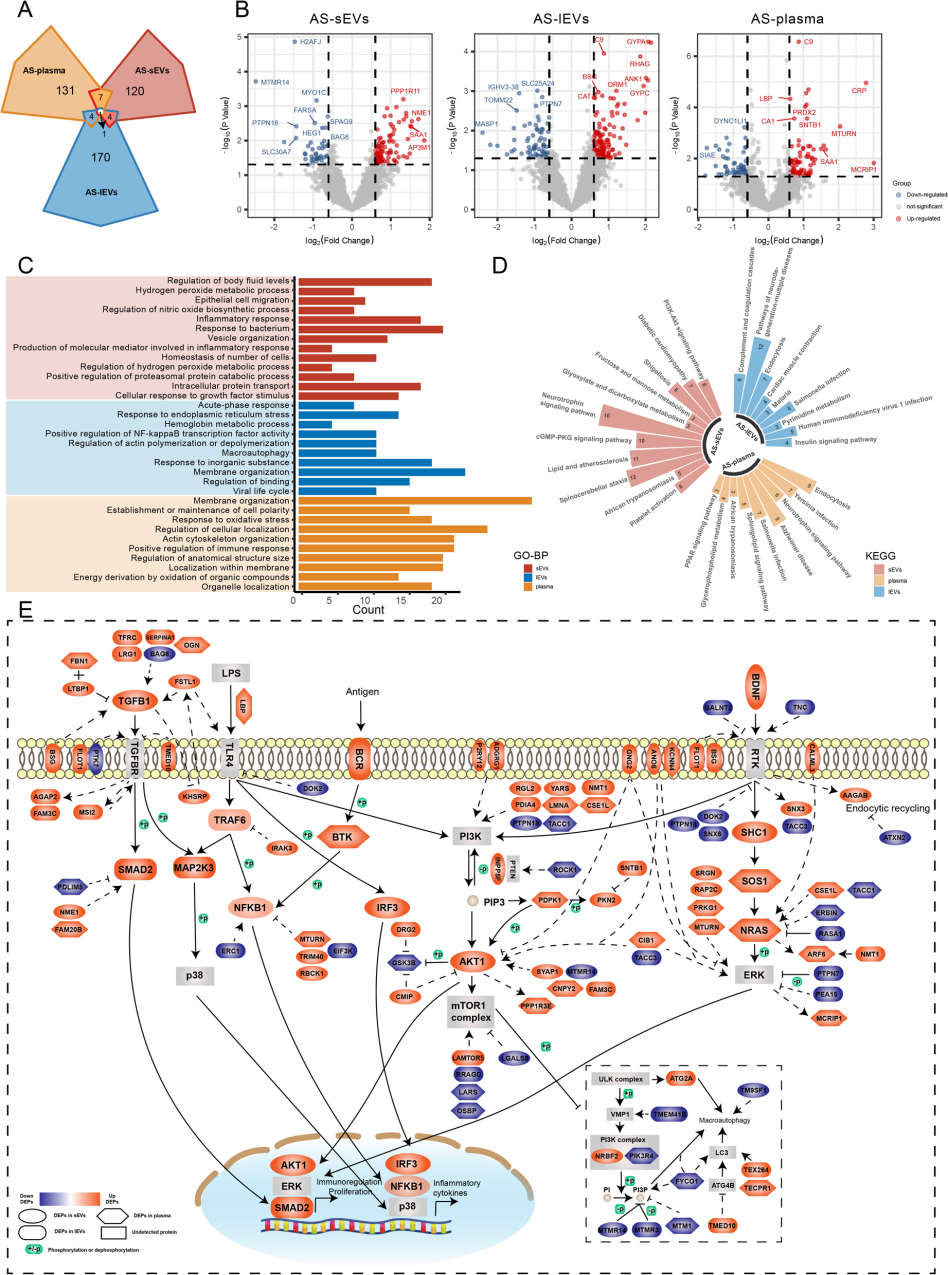
Proteomic landscape of plasma-derived EVs and plasma in AS.** A** Venn diagram showing the number of DEP overlaps for both EVs subpopulations and plasma in AS. **B** Volcano plots showing the distribution of DEPs in AS. **C** Bar plot displaying the GO analysis results according to the biological process category in AS. **D** Polar bar plot showing the pathway enrichment analysis results in AS using the KEGG database. **E** Overview of the proposed DEP-map of AS. *AS* ankylosing spondylitis-related acute anterior uveitis, *DEP* differentially expressed protein

For insight into the pathogenesis of the three types of uveitis and SCL, we performed detailed proteomic analyses for each disease in sEVs, lEVs, and plasma, independently. All four diseases were analyzed for comparison with HCs as a control group. The Venn diagram (Fig. 3A, Additional file 8: Table S3, and Additional file 2: Fig. S2, Additional file 3: Fig. S3, Additional file 4: Fig. S4: A) for each disease revealed a minor overlap of DEPs among sEVs, lEVs, and plasma. The top-ranked DEPs of the three components are presented in volcano plots (Fig. 3B, Additional file 8: Table S3, and Additional file 2: Fig. S2, Additional file 3: Fig. S3, Additional file 4: Fig. S4: B). GO analysis in the “biological process” category and KEGG enrichment analysis were performed and the results are presented by multicolor bar charts (Fig. 3C, Additional file 2: Fig. S2, Additional file 3: Fig. S3, Additional file 4: Fig. S4: C) and polar bar plots (Fig. 3D, Additional file 2: Fig. S2, Additional file 3: Fig. S3, Additional file 4: Fig. S4: D).

In AS, inflammatory responses, response to bacterium, cellular response to growth factor stimulus, and PI3K/Akt signaling pathways were specifically enriched in sEVs. Moreover, macroautophagy and positive regulation of NF-κB transcription factor activity were enriched in lEVs, and the positive regulation of immune response and response to oxidative stress were enriched in plasma. In BD, regulation of NF-κB signaling and stress-activated MAPK cascade were enriched in sEVs, whereas complement activation and protein K63-linked ubiquitination were enriched in lEVs, and immune effector process and regulation of autophagy in plasma. Additionally, the NOD-like receptor signaling pathway was enriched in both lEVs and plasma. In SCL, the protein folding and response to ER stress were enriched in sEVs, the formation of neutrophil extracellular traps (NETs) was enriched in lEVs, and the response to cytokine and chemokines signaling pathway was enriched in plasma. Moreover, the innate immune response was enriched in both lEVs and plasma. In VKH, the negative regulation of MAPK cascade and ubiquitin-mediated proteolysis were enriched in sEVs, while the NF-κB and NOD-like receptor signaling pathways were enriched in lEVs. In addition, protein dephosphorylation was enriched in the plasma.

Distinct DEPs and enrichment pathways for sEVs, lEVs and plasma in the same disease highlight their diverse roles and the necessity of collectively investigating them to achieve a more comprehensive understanding of the pathological changes in the disease. Based on the DEPs and annotations in the GO and KEGG analyses, the DEP-maps of AS, BD, SCL, and VKH were summarized and plotted (Fig. 3E and Additional file 2: Fig. S2, Additional file 3: Fig. S3, Additional file 4: Fig. S4: E). The DEP-maps were flameworked by the annotated pathways in GO and KEGG enrichment analysis and built on the mapping principles in the KEGG database to depict potential relationships between DEPs with enriched pathways. Solid lines represent known interactions referenced to the KEGG database, and dashed lines denote possible interactions supported by published research. Possible post-translational modifications derived from the KEGG database are also highlighted for illustrating the whole biological process (post-translational modifications were not a focus in this study). From the DEP-maps, it is possible to learn the changes in the activation of specific pathways in diseased group and the sites of DEPs may act. In summary, microbial infection, autophagy regulated by PI3K/Akt pathway, inflammation modulation related to NF-κB, TGF-β, and MAPK pathways were critical for disease development in AS; function of the ubiquitin–proteasome system and regulation of NF-κB and Wnt pathways were important for BD; activation of retinoic acid-inducible gene-I (RIG-I)-like receptor (RLR) signaling pathway, function of ER and NETs were vital for SCL; apoptosis, regulation of MAPK and JAK pathways and the effect of protein phosphatase were critical for VKH.

In addition, we found a larger number of intracellular and transmembrane proteins in EVs involved in vital biological processes compared to those in plasma. These proteins are important contributors to disease progression, highlighting the superior value of EVs in revealing the underlying pathological mechanisms. These results reveal the proteomic landscape of plasma-derived EVs and plasma in AS, BD, SCL, and VKH compared to HCs, suggesting the most relevant biological processes for the specific disease.

### Combined candidate biomarker panels of plasma and EVs improve diagnostic performance

**Fig. 4:**
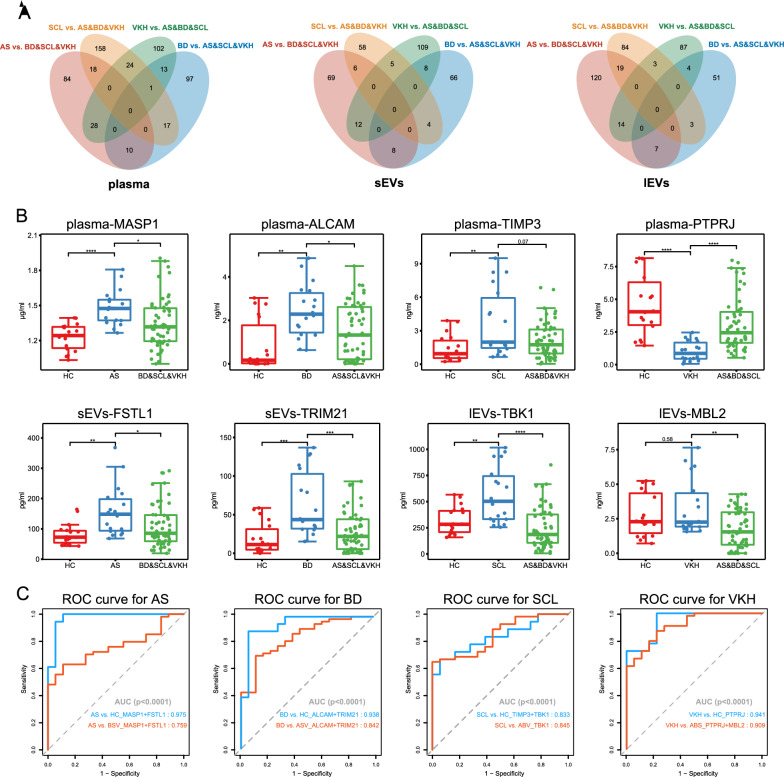
Combined candidate biomarker panels of plasma and EVs improve diagnostic performance.** A** Venn diagrams displaying the DEP overlaps (FC > 1.2, p < 0.04) between a single diseased group and the other three groups in plasma, sEVs, and lEVs. **B** Concentrations of defined biomarker candidates of MASP1, ALCAM, TIMP3, and PTPRJ in plasma, FSTL1 and TRIM21 in sEVs, and TBK1 and MBL2 in lEVs in the validation cohorts. The bottom, top, and middle of the boxplot represent the 25th, 75th percentile, and median, respectively; The whiskers below and above the boxplot indicate the 10th and 90th percentiles, respectively. **C** The AUC of the ROC of potential biomarker panels for the four diseases between a single diseased group versus the other three diseased groups or HCs in the validation cohort. *AS* ankylosing spondylitis-related acute anterior uveitis, *BD* Behcet's disease uveitis, *SCL* posterior scleritis, *VKH* Vogt-Koyanagi-Harada syndrome, *HC* healthy control, *ROC* receiver operating characteristic

The two EV subpopulations and plasma were selected as potential biomarker sources to distinguish between the four diseases. In the discovery phase, we performed a differential analysis between one disease set and the other three sets, followed by a Venn diagram analysis (Fig. 4A). Specifically, we selected the candidate biomarkers from the independent set of proteins in the Veen plot (Additional file 9: Table S4). Protein function was another key consideration for biomarker selection. We identified hub proteins using Cytohubba from independent sections of the Veen diagram and listed the top ten proteins in Additional file 5: Fig. S5. Finally, we chose MASP1 and MBL2 considered as hub proteins, ALCAM, TRIM21 and TIMP3 suggested by the previous research to be associated with the specific disease [28–30], and FSTL1, PTPRJ and TBK1 originated from DEP-maps for further ELISA validation (Fig. 4B). An independent cohort of 90 individuals, including 18 patients in each group (AS, BD, SCL, VKH, and HC) was enrolled (Additional file 6: Table S1), and 720 samples were measured. ROC analysis of the eight candidates between a single-disease group versus the others and HCs was conducted to assess differential diagnostic performance (Fig. 4C). The area under the curve (AUC) values of the eight candidates, MASP1, ALCAM, and PTPRJ in the plasma, FSTL1 and TRIM21 in sEVs, and TBK1 and MBL2 in lEVs, were > 0.8 in distinguishing a single-diseased group from HC. Similarly, the AUC values for distinguishing a single-diseased group from the other three diseased groups were > 0.75. Additionally, the diagnostic performance parameters, including cutoff point, sensitivity, specificity, positive likelihood ratio, negative likelihood ratio, positive predictive value, negative predictive value, and diagnostic odds ratio, are shown in Additional file 9: Table S4. These results demonstrate that the combined biomarker panels of plasma and EVs provide better diagnostic performance for these four diseases.

### Negative correlation between plasma ECE1 level and retinal thickness

**Fig. 5:**
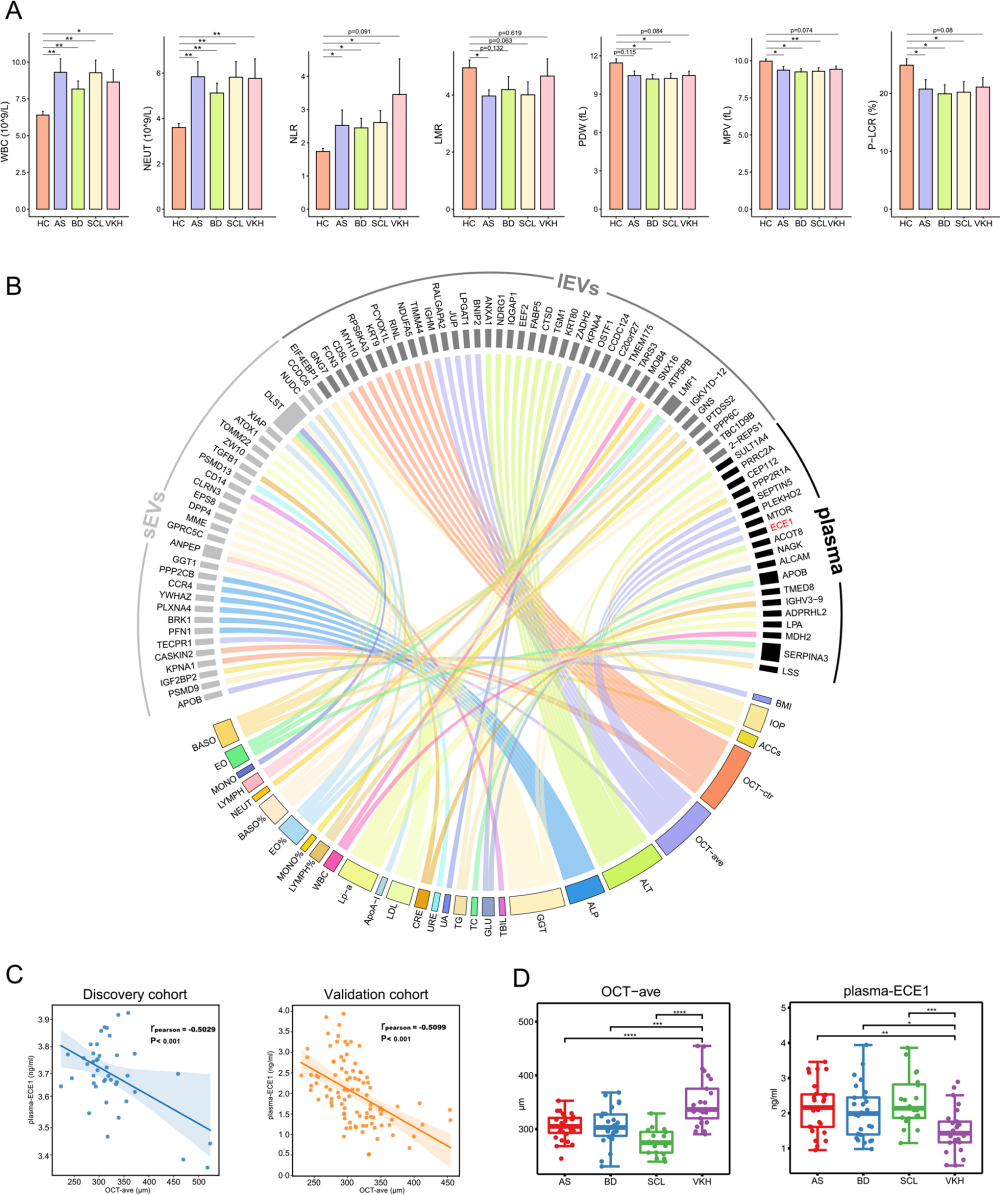
Negative correlation between plasma ECE1 level and retinal thickness. **A** The column charts indicate the value of WBC, NEUT, NLR, LMR, PDW, MPV, and P-LCR in five groups. **B** Chord diagram showing the relationships between clinical parameters and proteins in sEVs, lEVs, and plasma by Pearson correlation analysis. Only correlations with absolute values of correlation coefficients greater than 0.5 and p < 0.05 are displayed. **C** Linear fit was applied to the scatterplot in the discovery and validation cohorts. The Pearson correlation coefficient (r) and p-value (p) are displayed on each graph. **D** Boxplots showing the average thickness values of OCT and concentrations of ECE1 in plasma in the validation cohort. *WBC* white blood cells, *NEUT* neutrophil, *NLR* neutrophil-to-lymphocyte ratio, *LMR* lymphocyte to monocyte ratio, *PDW* platelet distribution width, *MPV* mean platelet volume, *P-LCR* platelet-large cell rate, *AS* ankylosing spondylitis-related acute anterior uveitis, BD Behcet's disease uveitis, *SCL* posterior scleritis, *VKH* Vogt-Koyanagi-Harada syndrome, *HC* healthy control, *OCT-ave* average retinal thickness on the optical coherence tomography scan, abbreviations of clinical parameters are listed in Additional file 10: Table S5

We collected and analyzed the laboratory findings and ophthalmologic parameters of 91 individuals (16AS/16BD/16SCL/11VKH/32HC) in the discovery cohort (Additional file 10: Table S5). Notably, 5 VKH patients were not included for this analysis because of the missing data. The results showed that white blood cell (WBC), neutrophil (NEUT), and monocyte (MONO) counts, red blood cell volume distribution width-CV (RDW-CV), platelet distribution width (PDW), platelet-large cell rate (P-LCR), mean platelet volume (MPV), neutrophil-to-lymphocyte ratio (NLR), and lymphocyte to monocyte ratio (LMR) significantly differed between the diseased group and HCs. Among them, WBC and NEUT counts were higher in four disease groups than those in HCs (p < 0.05, Fig. 5A). Moreover, the NLR was significantly higher in AS, BD, and SCL compared to HCs (p < 0.05). However, NLR was also higher in the VKH group (VKH vs. HC: 3.46 ± 3.56 vs. 1.74 ± 0.54), but with no statistical significance (p = 0.091). Notably, several parameters of platelet such as PDW, MPV, and P-LCR were significantly lower in the diseased group, with the VKH difference approaching statistical difference (p < 0.09). In addition, the platelet-to-lymphocyte ratio (PLR) was not found to be different between the diseased group and HCs. Multiple studies have demonstrated the ability of NLR and PLR for the prediction of uveitis occurrence and severity, with NLR always being more sensitive than PLR [31–33]. Therefore, the absence of a significant elevation of PLR in the diseased group in our study may be due to insufficient sample size. In our study, we found increased white blood cell differential counts, decreased platelet function indicators, and elevated inflammatory indicators in patients with uveitis and posterior scleritis, which are consistent with previous studies [31, 33, 34].

To improve the understanding of the molecular pathophysiology of clinical parameters in the diseased group, the correlations between proteomic profiles and biological clinical parameters were investigated by Pearson correlation analysis. In total, 89 proteins were strongly associated with 28 clinical parameters (absolute value of correlation coefficients > 0.5 and p < 0.05) (Fig. 5B). The association between various proteins and clinical parameters (i.e., BMI, liver and kidney function, blood lipids, complete blood, and ocular parameters) was found. These results provide interesting targets to explore the intrinsic mechanisms of change in clinical parameters, especially ocular parameters.

Of all ocular parameter-related proteins, a significant negative correlation (p < 0.001 r =  −  0.5029) between ECE1 in plasma and average retinal thickness on the optical coherence tomography OCT scan (OCT-ave) was detected (Fig. 5C). ECE1 is involved in the vasoregulation of the retina and has been demonstrated to be associated with retinal pigment epithelial (RPE) cell apoptosis [35, 36]. Therefore, we further validated the negative correlation (p < 0.001 r =  − 0.5099) between plasma ECE1 and OCT-ave values in an independent patient cohort (n = 98, Additional file 6: Table S1, Fig. 5C). Since multifocal exudative retinal detachment is one of the accompanying signs of VKH, a significantly higher OCT-ave was found in the VKH group compared to other groups in the discovery cohort (Additional file 6: Table S5). Correspondingly, in the validation cohort, the VKH group had higher OCT-ave and lower plasma ECE1 levels compared to the other groups (Fig. 5D).

### Prediction of potential therapeutic agents

**Fig. 6:**
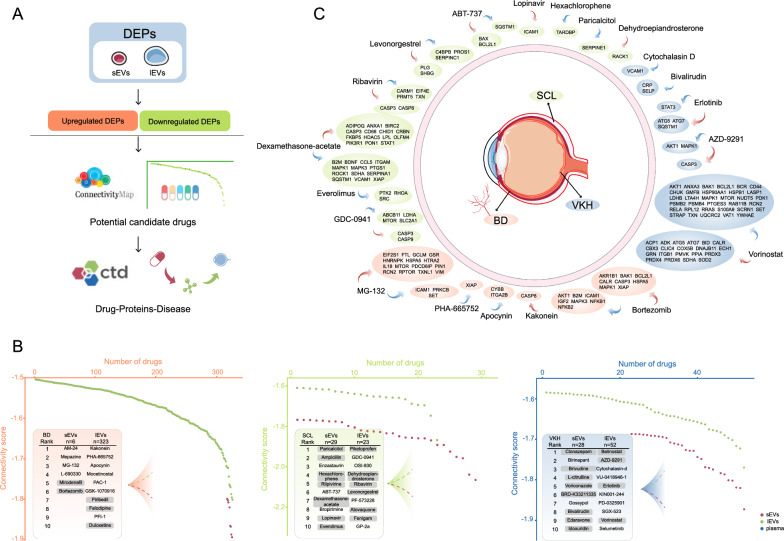
Prediction of potential therapeutic agents. **A** Workflow of drug prediction and analysis using online tools. **B** Rank plots showing the results of drug prediction in BD, SCL, and VKH, the top 10 drugs in sEVs, lEVs, and plasma were listed in the plots, and approved drugs for clinical use are in gray background. **C** Drug-protein-disease interactions were obtained through the CTD, the red arrows represent upregulated protein expression while the blue arrows represent downregulated protein expression. *BD* Behcet's disease uveitis, *SCL* posterior scleritis, *VKH* Vogt-Koyanagi-Harada syndrome

We performed drug prediction based on expression values of DEPs in two EV subgroups, followed by drug analysis using online websites, and the flow chart is shown in Fig. 6A. Since EVs were more relevant to immune disease than plasma, only EVs were included in this analysis. This analysis was not performed for the AS group as patients did not receive systemic treatment in this study. CMap was used to identify potential candidate drugs. Results were ranked based on the connectivity scores (Additional file 10: Table S6), and the top 10 drugs are listed in Fig. 6B. Agents that have already been launched are colored with grey background. Supplemental material lists the background and indication of the top 10 drugs shown in Fig. 6B according to the online chemical information websites (DrugBank, PubChem, and ChEBI). In addition, we retrieved the publications reporting the relationship between these drugs and immunity as well as inflammation (Additional file 12). Among these drugs, the following six categories are presented most frequently: proteasome inhibitors, HDAC inhibitor, MEK inhibitor, EGFR inhibitor, GABA receptor antagonist, and cytochrome P450 inhibitor.

Of these, dexamethasone-acetate has been used for the treatment of uveitis and scleritis, whereas bortezomib, everolimus, and vorinostat have also been investigated in animal models and patients with uveitis. In addition, multiple drugs have been reported to be effective in animal models of autoimmune diseases including experimental autoimmune encephalomyelitis and collagen-induced arthritis, such as mepazine, vorinostat, enzastaurin, bropirimine, belinostat, BRD-K33211335, gossypol, and edaravone. Moreover, BRD-K33211335 has been found to play an immunomodulatory role in systemic lupus erythematosus and rheumatoid arthritis.

The CTD was used to identify the drug–protein interaction network with the aim of helping us visualize and evaluate which signature proteins these drugs might target to affect uveitis and scleritis (Fig. 6C). According to the CTD, the possible targets of 20 agents for BD, SCL, and VKH were identified and the directions of drug activities were labeled.

## Discussion

Our data showed a relatively higher number of proteins (3,658) in EVs and plasma that can be quantified compared to those of previous studies [37, 38]. This is attributed to the following: (i) the large sample size and abundant disease types of the samples included in our study; (ii) the isolation method of EVs; and (iii) the construction of the IDA spectral library. Regarding the latter, we established an IDA spectral library containing up to 3,978 proteins using sEVs, lEVs, and plasma. The plasma is known to contain high-abundance proteins such as albumin, IgG, and lipoproteins, which mask the low-abundance proteins and result in a lower number of identified proteins in plasma during IDA. To build a larger spectral library, depletion of high-abundance proteins on plasma samples was performed. ExoCarta is an exosome protein, RNA, and lipid database, updated in 2015, that included 41,860 protein entries [39]. We screened 5403 exosomal proteins of homo sapiens from the Exocarta database and found that approximately 1000 additional exosomal proteins were identified in our study. Therefore, our results may also contribute to the enhancement of the current exosomal protein database as well as support future studies involving exosomes. By analyzing the proteomic profiles of sEVs, lEVs, and plasma, we explored the potential pathological mechanisms for uveitis and posterior scleritis.

AS is a relatively common autoimmune disease with numerous undefined etiological mechanisms. The contribution of *Klebsiella pneumoniae* infection to AS pathogenesis has been previously proposed and shown to provoke the inflammatory cascade and host tissue damage by inducing genetically susceptible individuals to produce antibodies that cross-react with host HLA-B27 epitopes and collagens [40, 41]. In addition, microbial antigens mediate HLA-B27-related diseases via the TLR pathway [42]. Viruses and bacteria maintain their survival and continuous replication by activating the PI3K/Akt pathway, followed by autophagy inhibition [43–45]. Autophagy is responsible for the degradation of misfolded HLA-B27 [46]. However, autophagy-related genes in PBMC of patients with AS were downregulated and positively correlated with disease severity [47]. Bacterial infections, particularly *Klebsiella*, might stimulate the TLR pathway, downstream interferon regulatory factor 3 (IRF3) molecules, and the NF-κB and PI3K/Akt pathways via molecular mimicry. The former two signaling pathways could be directly involved in the modulation of the inflammatory response, whereas the latter could mediate bacterial survival and HLA-B27 clearance through its effects on autophagy. Additionally, the TGF-β [48] and MAPK [49] signaling pathways have been implicated in the regulation of inflammation in AS. Of note, some key molecules in the abovementioned pathways, such as TGF-β, AKT1, IRF3, SMAD2, and MAP2K3, were identified in EVs but not in plasma. Therefore, the dysregulated proteins found in the EV fractions provided more critical information than the plasma fraction for a better understanding of the disease mechanisms of AS. The high expression of FSTL1 in sEVs was validated using ELISA in our study. FSTL1 is a pro-inflammatory molecule capable of facilitating pro-inflammatory cytokine production [50, 51]. Moreover, FSTL1 was expressed after *Streptococcus pneumoniae* infection, positively modulated the NLRP3 (NACHT, LRR, and PYD domain-containing protein 3) inflammasome, and promoted inflammatory injury during infection through the TLR4/NF-κB signaling pathway [52]. Therefore, we speculate that FSTL1 is closely associated with the etiology of infection and regulation of inflammation in AS, revealing a potential pathogenic mechanism and therapeutic target for AS.

BD is a chronic, recurrent, and remitting vasculitis with unclear etiology. Viral infections (e.g., herpes simplex virus [HSV]) and cross-reactivity between human and bacterial heat-shock proteins may contribute to the etiology of BD [53]. Combining our findings, we hypothesized that the activated TLR and NLR signaling pathways in patients with BD were potentially triggered by microbes, subsequently activating the NF-κB signaling cascade and the NLRP3 inflammasome, thereby setting an inflammatory response in motion. However, excessive NLRP3-mediated inflammation occurs in the presence of dysfunctional autophagy. A study on intestinal BD showed that defective autophagy is responsible for inflammasome activation and cell death [54]. In this study, the protein ubiquitination process was enriched in the plasma, whereas a variety of ubiquitinating enzymes were up-regulated in both sEVs and lEVs. The ubiquitin–proteasome system (UPS) is required for the degradation of damaged and misfolded proteins and participates in lymphocyte growth, activation, differentiation, and regulation of inflammatory factors [55]. Additionally, the UPS is a critical modulator of the Wnt and NF-κB signaling pathways through the degradation of β-catenin and IκBa [56], which could potentially be involved in regulating inflammatory processes and angiogenesis in BD [57–59]. Therefore, we speculate that functional enzymes involved in protein ubiquitination are present in circulating plasma or encapsulated in EVs, subsequently traveling to specific sites in the body to exert their effects and regulate intracellular signaling pathways. TRIM21, an E3 ubiquitin ligase that plays a myriad of cellular functions, including regulation of innate immune responses and anti-viral defense [28], was upregulated in sEVs of BD. In addition, TRIM21 was found to be upregulated in monocytes from BD patients and stimulated the NF-κB signaling pathway, leading to the production of monocyte-derived proinflammatory cytokines and the promotion of Th1/Th17-mediated inflammation [28]. The present study further highlights the vital role of TRIM21 in the pathogenesis of BD, perhaps with the aid of sEVs as carriers of intercellular transmission.

In most cases, SCL is caused by autoimmunity [60]. Our study revealed that protein processing in the ER and defense responses to the microbes are enriched in SCL. On the one hand, proteins involved in correct folding in the ER were identified as downregulated; on the other hand, proteins that respond to misfolding, such as those engaged in ER stress and ER-associated degradation, were up-regulated. These results indicate that excessive ER stress induced by misfolded proteins may occur in SCL, subsequently exacerbating inflammation. More importantly, the anti-microbial defense responses were markedly enriched in SCL. We identified several DEPs involved in the RIG-I-like receptor signaling pathway, which is typically employed to detect pathogenic RNAs and induce antiviral responses. Since the relationship between SCL and viral infection is unclear, molecular mimicry may be a potential trigger for autoimmune attacks. However, we speculate that the occurrence of SCL was not necessarily directly correlated with infection but may be due to failure in self and non-self-discrimination. Once self-RNAs are improperly masqueraded and recognized or single nucleotide polymorphism in the RLR gene contribute to the deletion of autoinhibition, the immune response is induced by RLRs [61]. For instance, mutations in RLR-related genes in systemic lupus erythematosus led to multiorgan damage triggered by hyperactive lymphocytes via the enhanced production of autoantibodies against their nucleic acids and RNA-binding proteins [62]. SCL may have similar intrinsic patterns. Additionally, NETs, which serve as a potential source of autoantigens, may break self-tolerance and aggravate the production of autoantibodies in a positive feedback manner in SCL [63]. TBK1, a critical mediator in the RIG-I signaling pathway [64], was upregulated in the lEVs of SCL. Accumulating evidence suggested that self-DNA or RNA activates TBK1 kinase activity in immune cells [64]. We suspected that TBK1 is involved in RLR-mediated signaling activated by an undefined stimulus, as well as downstream IFN-I in SCL. Significant developments have been made in recent years regarding TBK1 inhibitors that can effectively ameliorate autoimmune and inflammatory diseases in animal models, some of which are already in or about to enter clinical trials [65]. Future intensive research into SCL holds the promise of including TBK1 inhibitors in treatment.

A T cell-mediated autoimmune process directed against one or more antigenic components of melanocytes is involved in VKH [66]. The programmed cell death (apoptosis) of lymphocytes is critical for maintaining immunological homeostasis and self-tolerance. Aberrant apoptosis can lead to autoimmune disorders. Studies have found that lymphocytes in patients with VKH are relatively resistant to apoptosis mediated by anti-Fas antibodies [67]. Moreover, higher Bcl-2 expression in CD4^+^ T cells is involved in the regulation of apoptosis of inflammatory cells in the cerebrospinal fluid of VKH patients [68]. In addition, the FAS gene copy number was associated with an increased risk of VKH in Han Chinese [69]. Accordingly, we speculate that the ERK/MAPK, PI3K/Akt, and NF-κB signaling pathways contribute to the proliferation and survival of lymphocytes, especially T cells, in VKH. However, the c-Jun JNK/MAPK, P38/MAPK, and JAK/STAT signaling pathways are considered to be involved in the development of inflammatory responses. Several proteins with dephosphorylation activity, such as tyrosine-protein phosphatase non-receptor type 1 (PTPN1) and PTPRJ, have been identified in both the circulating plasma and EVs. The downregulation of PTPRJ in the plasma was validated in VKH by ELISA. PTPRJ is a receptor-like protein tyrosine phosphatase that plays a role in the regulation of cell proliferation, migration, and differentiation by acting on the dephosphorylation of a variety of substrates, such as epidermal growth factor receptor, MAPK, T-cell receptor (TCR), and JAK2 [70, 71]. Therefore, we speculate that PTPRJ deficiency promotes the activation of the ErbB, JAK, and TCR signaling pathways in VKH, leading to apoptosis inhibition and proliferation induction, especially in immune cells. These dephosphorylating proteins potentially contribute to the modulation of inflammatory activation and immune cell proliferation, suggesting a novel potential intervention target for VKH.

Furthermore, we investigated the correlation of clinical measurements with protein expression profiles and demonstrated a negative relationship between OCT-ave and plasma ECE1 levels. ECE1 is a protease that cleaves big endothelin‐1 (ET1) into its active form, promoting vasoconstriction. The role of ECE1 in vasoregulation has been demonstrated in the blood vessels of the retina, optic nerve, and choroid [35]. In carcinoma cells and ventricular myocytes, downregulation of ECE1 resulted in cell apoptosis [72, 73]. Importantly, RPE cell apoptosis caused by mechanical stretching could be mediated by effects on the actin cytoskeleton and subsequent downregulation of ECE1 [36]. Therefore, low expression of ECE1 might lead to disruption of the blood–eye barrier, exudation, and detachment of the neuroepithelial layer by inducing apoptosis of RPE cells and vasodilation. These intrinsic reasons may explain the negative correlation between ECE1 expression levels and mean retinal thickness. This result suggested that the expression level of ECE1 could be used as an indicator of the average retinal thickness measured by OCT, thus reflecting the severity of exudative retinal detachment (ERD), and could also be used as a therapeutic target for ERD and a diagnostic biomarker for VKH.

In addition to first-line glucocorticoid therapy, a better and more effective therapeutic approach for uveitis and posterior scleritis requires continuous exploration. The potential immunomodulatory activity of histone deacetylase inhibitors (HDACis) makes them interesting therapeutic candidates for the treatment of a variety of human diseases, such as cancer, autoimmune, and inflammatory diseases [74]. Three HDACis predicted in this study, such as mocetinostat, vorinostat, and belinostat, are considered to have potential for the treatment of BD and VKH. Notably, vorinostat and belinostat were approved for clinical use, and their further investigation for the treatment of uveitis would be meaningful. Proteasome inhibitors have been successfully developed as a therapy for patients with autoimmune diseases and immune-mediated disorders. Interestingly, two proteasome inhibitors, bortezomib and MG-132, were identified for the treatment of BD, suggesting a significant role of protein ubiquitination in the pathogenesis of BD, which is also consistent with the aforementioned findings. In SCL, multiple antiviral and fungal agents, such as ampicillin, rilpivirine, lopinavir, ribavirin, and atovaquone were predicted to be effective, implying that infection could be the key biological process during disease progression. As mentioned previously, we highlighted the fact that apoptosis might be critical for the development of VKH. Meanwhile gossypol, an inhibitor of the apoptosis-inhibiting protein BCL2, offers the opportunity to treat VKH by modulating this process. In conclusion, these drugs could be validated in future trials as our results have shown their relevance to the intrinsic pathological mechanisms of disease and their potential for uveitis and scleritis treatment.

This study has few limitations. First, all enrolled patients were in the acute or active inflammatory phase, restricting the application range of the biomarkers. It would be ideal to involve more patients from a wider range of disease periods to explore specific biomarkers for the disease. Second, the proposed potential biological processes were not validated. Although we confirmed some critical dysregulated proteins in the DEP-map using ELISA, their detailed roles in the pathogenesis of uveitis and SCL require further investigation. Finally, the potential biomarkers identified in our study offer insights for subsequent research, but validation in larger populations remains necessary.

## Conclusion

Overall, we present a systematic proteomic investigation of plasma-derived sEVs, lEVs, and plasma in patients with four types of ocular immune diseases and HCs. We established an IDA library using EVs and plasma, which greatly improved the number of proteins identified. The proteomic profiles of sEVs, lEVs, and plasma were completely different for the same disease, as suggested by their DEPs and the enriched pathways involved. Therefore, investigations of all three components are necessary to obtain a comprehensive picture of the diseases. We identified potential biomarker panels for each disease and analyzed the correlation between clinical parameters and proteomic data. Furthermore, we predicted potential therapeutic agents for systemic treatment of diseases. In conclusion, our study serves as a valuable informational resource for future proteomic studies to better understand uveitis and posterior scleritis, provides new insights into their possible pathogenesis, and identifies novel potential biomarker panels and therapeutic agents.

## Supplementary Information

**Additional file 1: Fig. S1. **Isolation and characterization of plasma-derived EVs and proteomic analysis, related to Figs. 1, 2.** A** Schematic diagram of the sEV and lEV isolation procedure. **B** Representative nanoparticle tracking analysis plots of sEVs and lEVs. **C **Transmission electron microscopy images of sEVs and lEVs. **D** Relative grayscale of western blot analysis shows the presence of EV markers, Grp94 in lEVs, and Apo-A1 as a negative purity control. **E** Coomassie brilliant blue staining showing the distribution of protein content in sEVs, lEVs, and plasma.** F** Violin plot of the coefficient of variation for quality control; Histograms showing the distribution of coefficient of variation in sEVs, lEVs and plasma samples. **G** Venn diagrams showing the overlap of quantifiable protein between sEVs and lEVs samples. **H** Venn diagrams showing the overlap of quantifiable protein between sEVs, lEVs, and plasma samples. **I** Balloon plot showing the VIP scores in the PLS-DA model for the three components, and the VIP scores are presented as the average scores of PLS-DA components. **J **Venn diagrams showing the overlap of significantly downregulated DEPs in the four diseases among the three components and the overlap of downregulated DEPs in the three components among the four diseases. *AS* ankylosing spondylitis-related acute anterior uveitis, *BD* Behcet's disease uveitis, *SCL* posterior scleritis, *VKH* Vogt-Koyanagi-Harada syndrome, *HC* healthy control, *DEP* differentially expressed proteins.

**Additional file 2: Fig. S2.** Proteomic landscape of plasma-derived EVs and plasma in BD. **A** Venn diagram showing the number of DEP overlaps for both EV subpopulations and plasma in BD. **B** Volcano plots showing the distribution of DEPs in BD. **C** Bar plot displaying the GO analysis results according to the biological process category in BD. **D** Polar bar plot showing the pathway enrichment analysis results in BD using the KEGG database. **E** Overview of the proposed DEP-map of BD. *BD* Behcet's disease uveitis.

**Additional file 3: Fig. S3. **Proteomic landscape of plasma-derived EVs and plasma in SCL. **A** Venn diagram showing the number of DEP overlaps for both EV subpopulations and plasma in SCL. **B** Volcano plots showing the distribution of DEPs in SCL. **C** Bar plot displaying the GO analysis results according to the biological process category in SCL. **D** Polar bar plot showing the pathway enrichment analysis results in SCL using the KEGG database. **E** Overview of the proposed DEP-map of SCL. *SCL* posterior scleritis.

**Additional file 4: Fig. S4.** Proteomic landscape of plasma-derived EVs and plasma in VKH. **A** Venn diagram showing the number of DEP overlaps for both EV subpopulations and plasma in VKH. **B** Volcano plots showing the distribution of DEPs in VKH. **C** Bar plot displaying the GO analysis results according to the biological process category in VKH. **D** Polar bar plot showing the pathway enrichment analysis results in VKH using the KEGG database. **E** Overview of the proposed DEP-map of VKH. *VKH* Vogt-Koyanagi-Harada syndrome.

**Additional file 5: Fig. S5. **Top ten hub proteins in sEVs, lEVs, and plasma for specific disease, related to Fig. 4. **A** Hub proteins network in sEVs, lEVs, and plasma for AS vs. BD/SCL/VKH. **B** Hub proteins network in sEVs, lEVs, and plasma for BD vs. AS/SCL/VKH. **C** Hub proteins network in sEVs, lEVs, and plasma for SCL vs. AS/BD/VKH. **D** Hub proteins network in sEVs, lEVs, and plasma for VKH vs. AS/BD/SCL. *AS* ankylosing spondylitis-related acute anterior uveitis, *BD* Behcet's disease uveitis, *SCL* posterior scleritis, *VKH* Vogt-Koyanagi-Harada syndrome.

**Additional file 6: Table S1.** Descriptive statistics of the discovery and validation cohorts and list of identified and quantifiable proteins.

**Additional file 7: Table S2.** Related to Fig. 1B-C and Additional file 1: Fig. S1G-H. List of proteins in the Venn diagrams.

**Additional file 8: Table S3.** Related to Fig. 2. List of differentially expressed proteins for comparison between groups in sEVs, lEVs, and plasma.

**Additional file 9: Table S4.** Related to Fig. 4C. Diagnostic performance of biomarker panels for AS, BD, SCL, and VKH.

**Additional file 10: Table S5.** Related to Fig. 5A. Clinical parameters of the discovery cohort.

**Additional file 11: Table S6.** Related to Fig. 6B-C. List of predicted drugs by CMap and terms in the drug-protein interaction network by CTD.

**Additional file 12.** Results of drug prediction for BD, SCL and VKH.

## Data Availability

The mass spectrometry proteomics data have been deposited to the ProteomeXchange Consortium (http //proteomecentral.proteomexchange.org) with the dataset identifier PXD036844. Additional figures and tables are present in the accompanying supplemental documentation.

## Abbreviations

ALCAM: Activated leukocyte cell adhesion molecule
AS: Ankylosing spondylitis
AUC: Area under the curve
BD: Behcet's disease
BMI: Body mass index
CBB: Coomassie brilliant blue staining
CMap: Connectivity map
CTD: Comparative Toxicogenomics Database
DEPs: Differentially expressed proteins
ECE1: Endothelin-converting enzyme 1
ELISA: Enzyme-linked immunosorbent assay
ER: Endoplasmic reticulum
ERD: Exudative retinal detachment
ET1: Endothelin‐1
EVs: Extracellular vesicles
FC: Fold change
FDR: False discovery rate
FSTL1: Follistatin-related protein 1
GO: Gene Ontology
GSEA: Gene set enrichment analysis
GSVA: Gene set variation analysis
HC: Healthy control
HLA: Human leukocyte antigen
HSV: Herpes simplex virus
IDA: Information-dependent acquisition
IRF3: Interferon regulatory factor 3
ISEV: International Society for Extracellular Vesicles
KEGG: Kyoto Encyclopedia of Genes and Genomes
MASP1: Mannan-binding lectin serine protease 1
MBL2: Mannose-binding protein C
NETs: Neutrophil extracellular traps
NTA: Nanoparticle tracking analysis
OCT: Optical coherence tomography
PBMC: Peripheral blood mononuclear cells
PBS: Phosphate-buffered saline
PCA: Principal component analysis
pSCL: Posterior scleritis
PTPRJ: Receptor-type tyrosine-protein phosphatase eta
RIG-I: Retinoic acid-inducible gene-I
RPE: Retinal pigment epithelial
SWATH-MS: Sequential window acquisition of all theoretical fragment ion spectra-mass spectrometry
TBK1: TANK-binding kinase 1
TEM: Transmission electron microscopy
TIMP3: Metalloproteinase inhibitor 3
TRIM21: Tripartite motif 21
UPS: Ubiquitin–proteasome system
VIP: Variable importance in projection
VKH: Vogt-Koyanagi-Harada syndrome
