# Supplementary figures and images for: Comprehensive profiling of extracellular vesicles in uveitis and scleritis enables biomarker discovery and mechanism exploration
Source: J Transl Med. 2023 Jun 15;21:388. doi: 10.1186/s12967-023-04228-x (PMC10273650; doi:10.1186/s12967-023-04228-x)

# Additional file 1

A

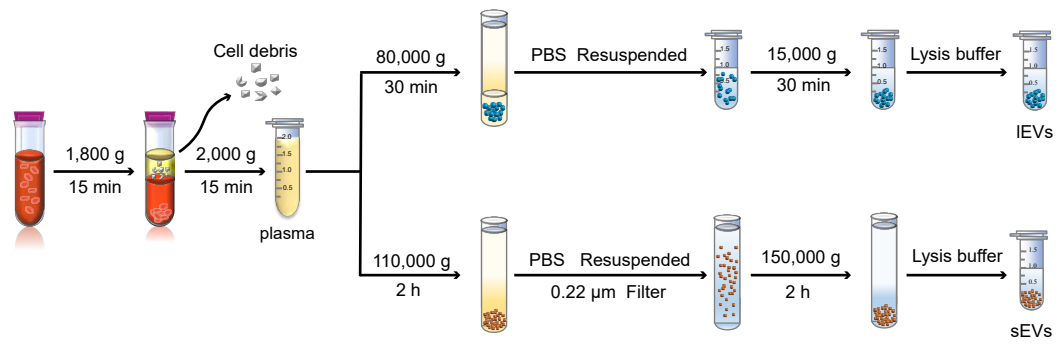

B

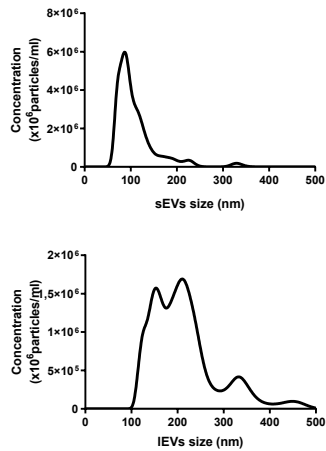

C

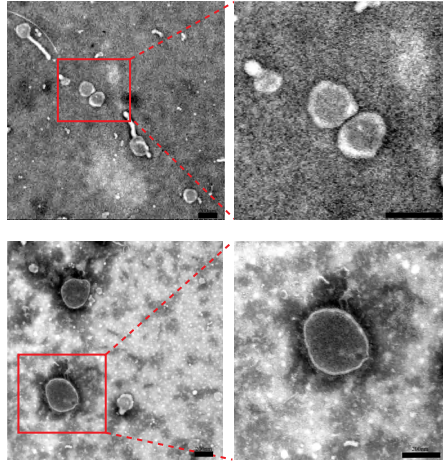

D

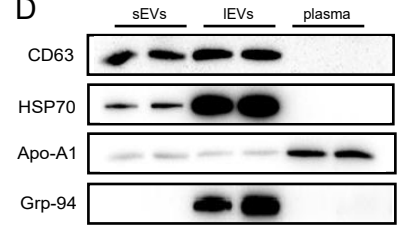

E

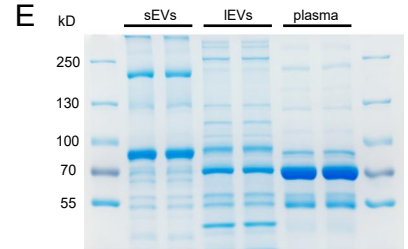

F

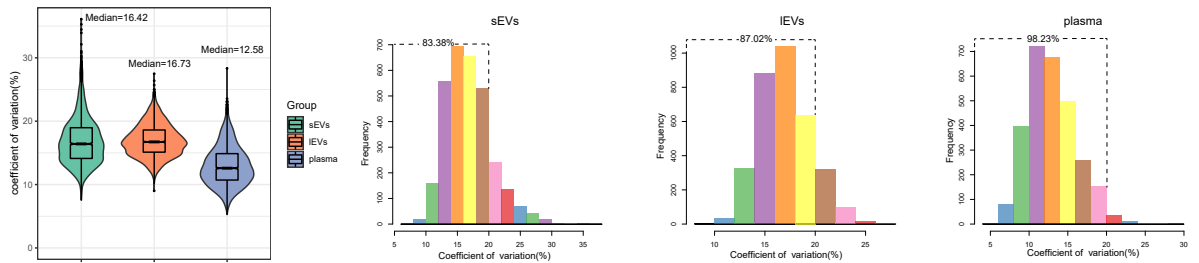

G

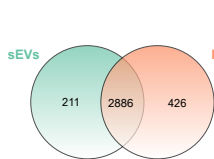

H

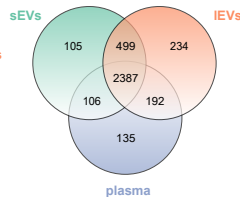

I

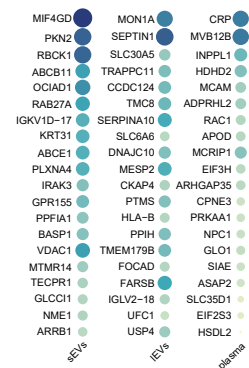

J

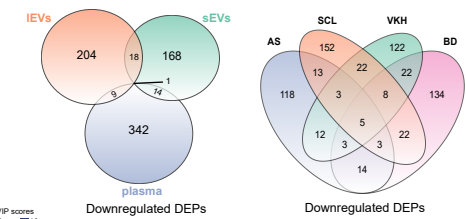

Supplement: Supplementary file 1 — Additional file 1: Fig. S1. Isolation and characterization of plasma-derived EVs and proteomic analysis, related to Figs. 1, 2. A Schematic diagram of the sEV and lEV isolation procedure. B Representative nanoparticle tracking analysis plots of sEVs and lEVs. C Transmission electron microscopy images of sEVs and lEVs. D Relative grayscale of western blot analysis shows the presence of EV markers, Grp94 in lEVs, and Apo-A1 as a negative purity control. E Coomassie brilliant blue staining showing the distribution of protein content in sEVs, lEVs, and plasma. F Violin plot of the coefficient of variation for quality control; Histograms showing the distribution of coefficient of variation in sEVs, lEVs and plasma samples. G Venn diagrams showing the overlap of quantifiable protein between sEVs and lEVs samples. H Venn diagrams showing the overlap of quantifiable protein between sEVs, lEVs, and plasma samples. I Balloon plot showing the VIP scores in the PLS-DA model for the three components, and the VIP scores are presented as the average scores of PLS-DA components. J Venn diagrams showing the overlap of significantly downregulated DEPs in the four diseases among the three components and the overlap of downregulated DEPs in the three components among the four diseases. AS ankylosing spondylitis-related acute anterior uveitis, BD Behcet's disease uveitis, SCL posterior scleritis, VKH Vogt-Koyanagi-Harada syndrome, HC healthy control, DEP differentially expressed proteins. [file 12967_2023_4228_MOESM1_ESM.pdf]

# Additional file 2

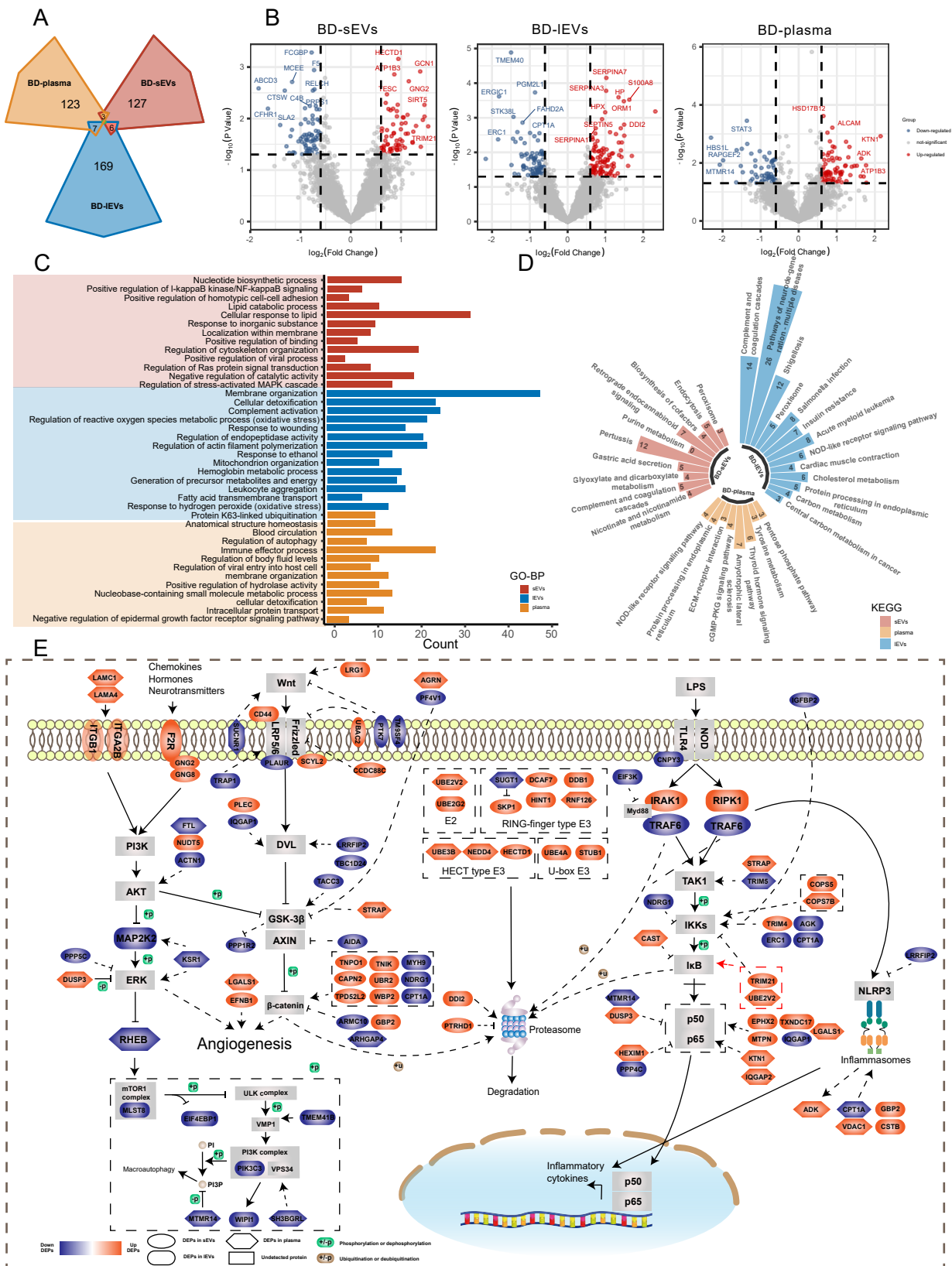

Supplement: Supplementary file 2 — Additional file 2: Fig. S2. Proteomic landscape of plasma-derived EVs and plasma in BD. A Venn diagram showing the number of DEP overlaps for both EV subpopulations and plasma in BD. B Volcano plots showing the distribution of DEPs in BD. C Bar plot displaying the GO analysis results according to the biological process category in BD. D Polar bar plot showing the pathway enrichment analysis results in BD using the KEGG database. E Overview of the proposed DEP-map of BD. BD Behcet's disease uveitis. [file 12967_2023_4228_MOESM2_ESM.pdf]

# Additional file 3

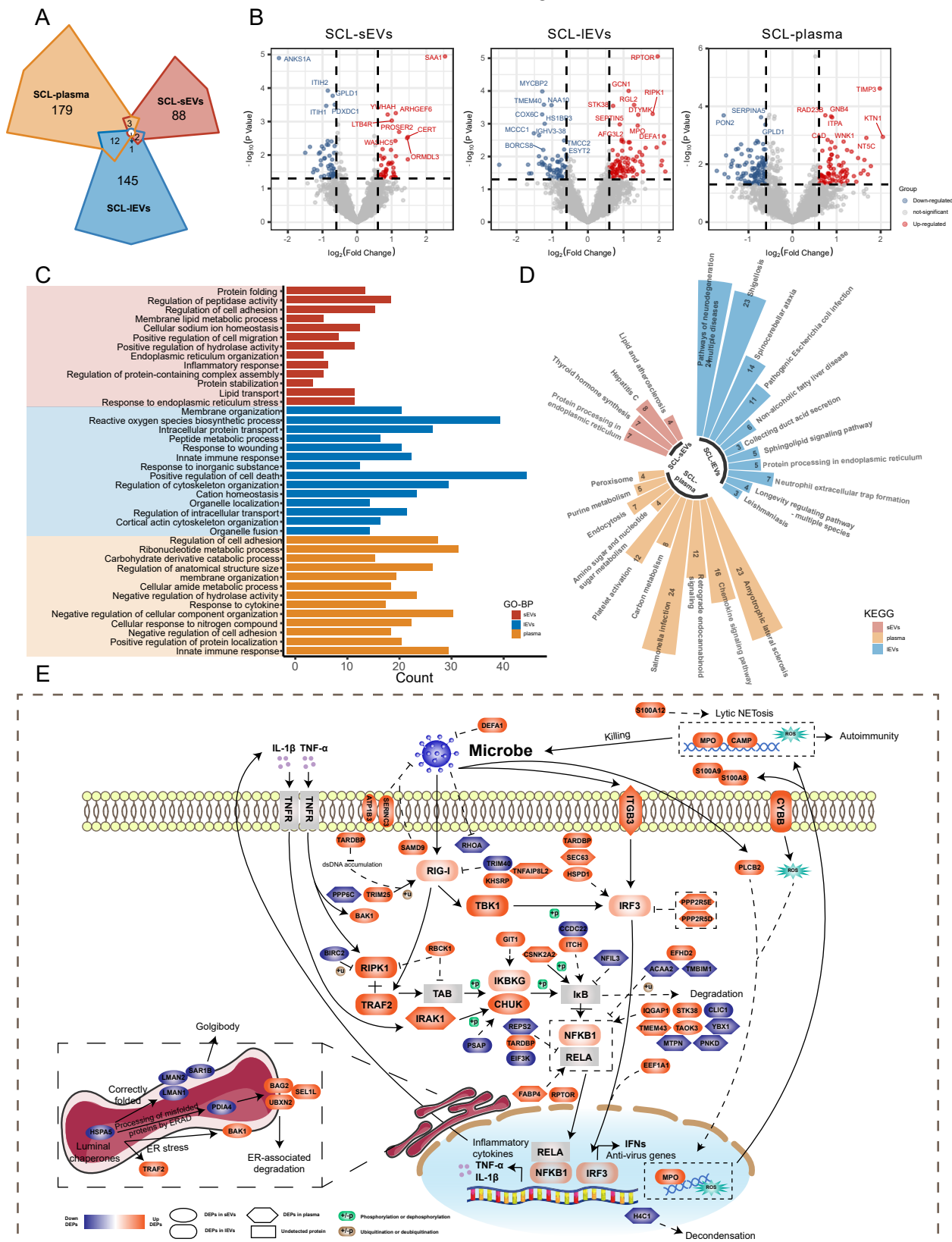

Supplement: Supplementary file 3 — Additional file 3: Fig. S3. Proteomic landscape of plasma-derived EVs and plasma in SCL. A Venn diagram showing the number of DEP overlaps for both EV subpopulations and plasma in SCL. B Volcano plots showing the distribution of DEPs in SCL. C Bar plot displaying the GO analysis results according to the biological process category in SCL. D Polar bar plot showing the pathway enrichment analysis results in SCL using the KEGG database. E Overview of the proposed DEP-map of SCL. SCL posterior scleritis. [file 12967_2023_4228_MOESM3_ESM.pdf]

# Additional file 4

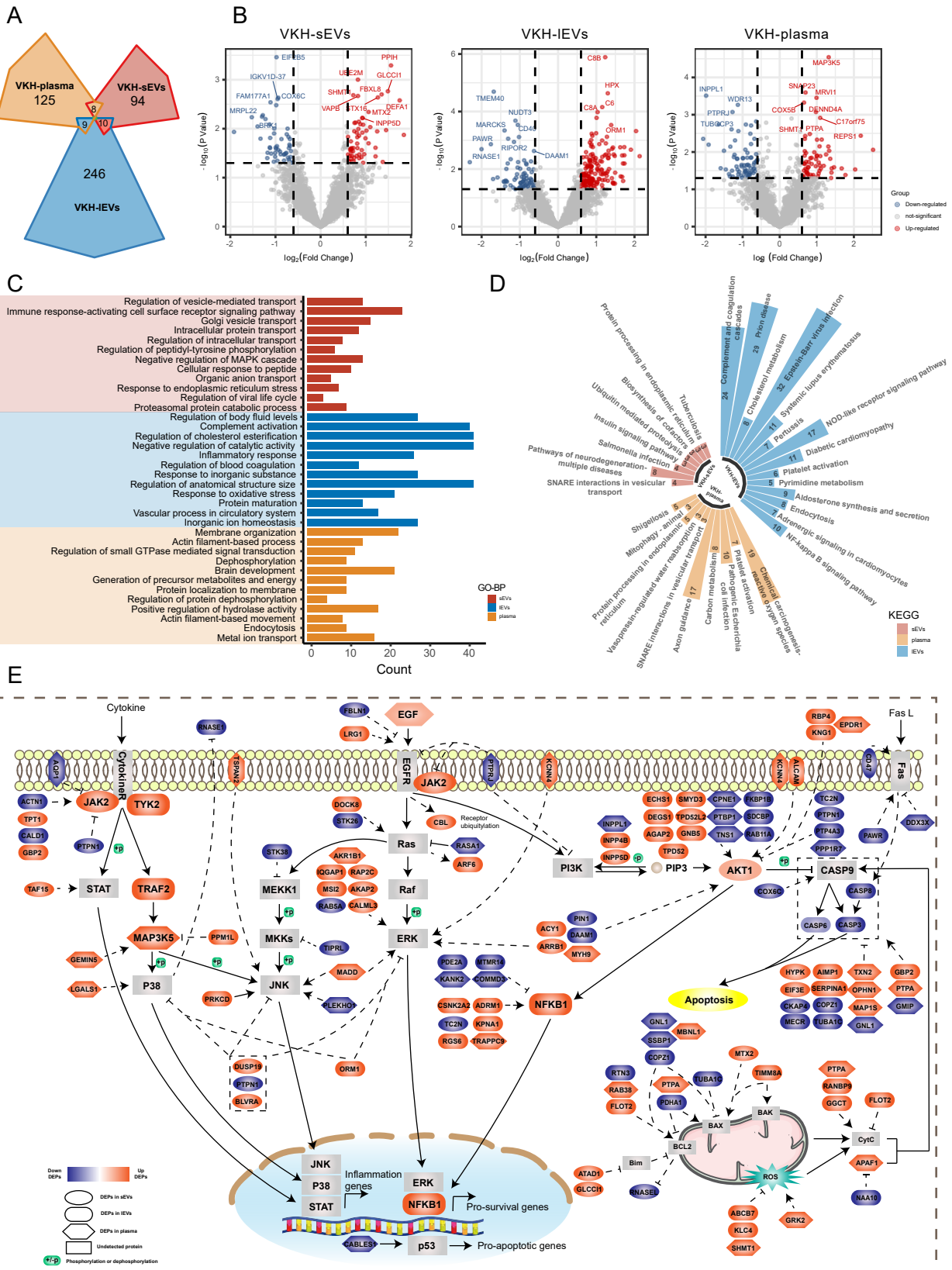

Supplement: Supplementary file 4 — Additional file 4: Fig. S4. Proteomic landscape of plasma-derived EVs and plasma in VKH. A Venn diagram showing the number of DEP overlaps for both EV subpopulations and plasma in VKH. B Volcano plots showing the distribution of DEPs in VKH. C Bar plot displaying the GO analysis results according to the biological process category in VKH. D Polar bar plot showing the pathway enrichment analysis results in VKH using the KEGG database. E Overview of the proposed DEP-map of VKH. VKH Vogt-Koyanagi-Harada syndrome. [file 12967_2023_4228_MOESM4_ESM.pdf]

A

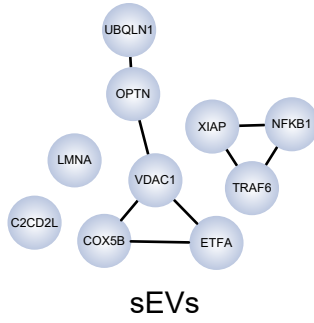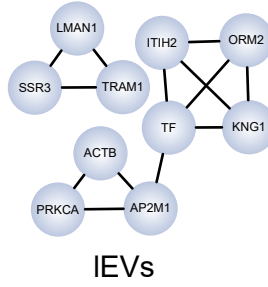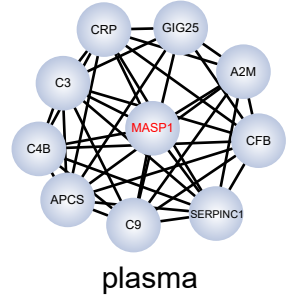

B

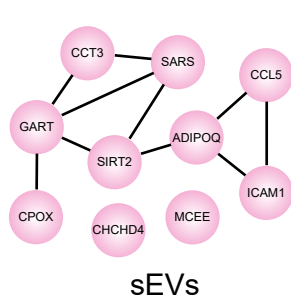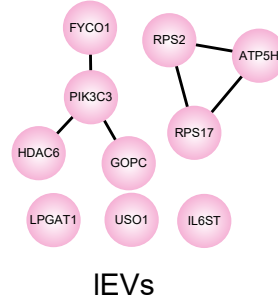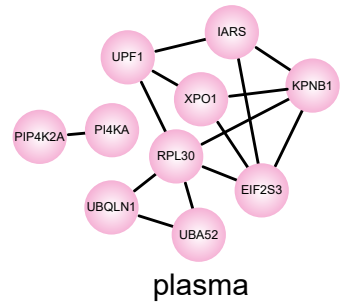

C

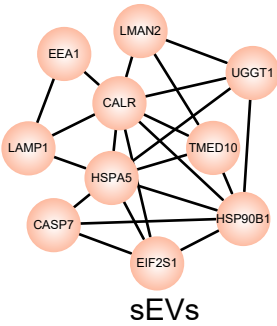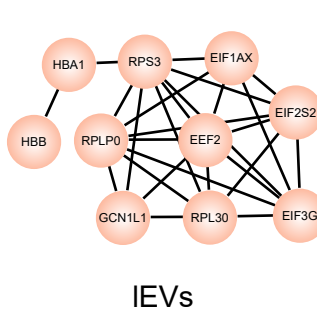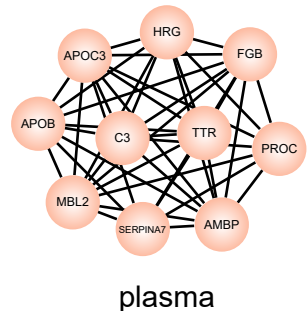

D

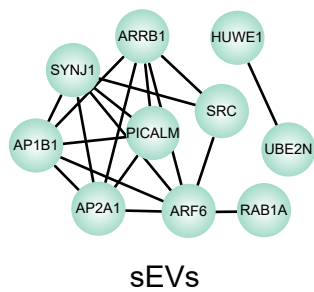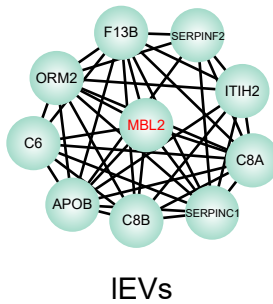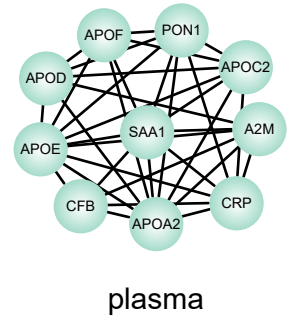

Supplement: Supplementary file 5 — Additional file 5: Fig. S5. Top ten hub proteins in sEVs, lEVs, and plasma for specific disease, related to Fig. 4. A Hub proteins network in sEVs, lEVs, and plasma for AS vs. BD/SCL/VKH. B Hub proteins network in sEVs, lEVs, and plasma for BD vs. AS/SCL/VKH. C Hub proteins network in sEVs, lEVs, and plasma for SCL vs. AS/BD/VKH. D Hub proteins network in sEVs, lEVs, and plasma for VKH vs. AS/BD/SCL. AS ankylosing spondylitis-related acute anterior uveitis, BD Behcet's disease uveitis, SCL posterior scleritis, VKH Vogt-Koyanagi-Harada syndrome. [file 12967_2023_4228_MOESM5_ESM.pdf]
