# Supplementary material for: Comprehensive profiling of extracellular vesicles in uveitis and scleritis enables biomarker discovery and mechanism exploration
Source: J Transl Med. 2023 Jun 15;21:388. doi: 10.1186/s12967-023-04228-x (PMC10273650; doi:10.1186/s12967-023-04228-x)
Supplement: Supplementary file 12 — Additional file 12. Results of drug prediction for BD, SCL and VKH. [file 12967_2023_4228_MOESM12_ESM.docx]

**Additional file 12**

**Comprehensive profiling of extracellular vesicles in uveitis and scleritis enables biomarker discovery and mechanism exploration**

Lingzi Wu^1,6^, Lei Zhou^2,3,6^, Jinying An^1,6^, Xianfeng Shao^4^, Hui Zhang^1^, Chunxi Wang^1^, Guixia Zhao^5^, Shuang Chen^1^, Xuexue Cui^1^, Xinyi Zhang^1^, Fuhua Yang^1^, Xiaorong Li^1^, Xiaomin Zhang^1,^*

***^1^****Tianjin Key Laboratory of Retinal Functions and Diseases, Tianjin Branch of National Clinical Research Center for Ocular Disease, Eye Institute and School of Optometry, Tianjin Medical University Eye Hospital, Tianjin, China;*

*^2^School of Optometry; Department of Applied Biology and Chemical Technology; Research Centre for SHARP Vision (RCSV); The Hong Kong Polytechnic University, Hong Kong, China*

*^3^Centre for Eye and Vision Research (CEVR), 17W Hong Kong Science Park, Hong Kong, China*

***^4^****State Key Laboratory of Proteomics, National Center for Protein Sciences (Beijing), Beijing Proteome Research Center, Beijing Institute of Lifeomics, Beijing, China;*

***^5^****Tangshan Eye Hospital, Tangshan, China.*

*Corresponding author. Tianjin Key Laboratory of Retinal Functions and Diseases, Tianjin Branch of National Clinical Research Center for Ocular Disease, Eye Institute and School of Optometry, Tianjin Medical University Eye Hospital, Tianjin, 300384, China.

E-mail address: [xzhang08@tmu.edu.cn](mailto:xzhang08@tmu.edu.cn) (X.Zhang)

^6^These authors contributed equally

| **Table 1 in Additional file 12. Results of drug prediction for BD.** | | | | |
| --- | --- | --- | --- | --- |
| Drugs | Status | Mechanism of action | Relationship with immunity and inflammation | Reference |
| AM-24 | Phase 2 | Lipoxygenase inhibitor | - AM-24 was described as a non-steroid anti-inflammatory molecule, dual inhibitor of cyclooxygenase and 5-lipoxygenase | [1] |
| Mepazine | Phase 2 | MALT1 inhibitor | - Administration of mepazine prophylactically or after disease onset attenuated EAE, while did not affect Treg development | [2] |
|  |  |  | - Mepazine suppressed human myeloid DC, effector T-cell, and B-cell responses, and retains Th1/Treg homeostasis | [3] |
| MG-132 | Preclinical | Proteasome inhibitor | - MG-132 suppressed the NF-κB-dependent proinflammatory gene expression in human RPE cells | [4] |
|  |  |  | - The combination of MG132 with rapamycin suppressed the expression of the inflammatory cytokines and the formation of macrophage foam cells and the activation of NF-κB pathway | [5] |
|  |  |  | - In vivo MG132 administration to atopic dermatitis mouse model reduced Th17 cells but maintained the level of Th1 cells | [6] |
| L-690330 | Preclinical | Inositol monophosphatase inhibitor |  |  |
| Bortezomib | Launched | NF-κB pathway inhibitor  proteasome inhibitor | - Bortezomib has been used to treat multiple myeloma and mantle cell lymphoma | DrugBank |
|  |  |  | - Bortezomib suppressed NF-κB activation and ameliorated eye inflammation in EAU | [7] |
|  |  |  | - Pretreatment with high-dose bortezomib significantly attenuated the inflammatory response of EIU by inhibiting the activation of NF-κB | [8] |
| Kakonein | Phase 2 | GABA receptor antagonist  serotonin receptor antagonist | - Puerarin has been investigated for the treatment of alcohol abuse | DrugBank |
|  |  |  | - Puerarin exhibited anti-inflammatory properties in acute lung injury rat model via regulation of the NF-κB signaling pathway | [9] |
| PHA-665752 | Preclinical | c-Met inhibitor | - PHA-665752 inhibited or prevented the proliferation of neoplasms | CHEBI |
|  |  |  | - T-cell infiltration was reduced in the hearts of PHA-665752-treated mice, particularly in the CD8 T-cell subset | [10] |
|  |  |  | - PHA-665752 treatment of tumor-derived T lymphocytes abolished phosphorylation of Met and downstream effectors and led to caspase-mediated apoptosis | [11] |
| Apocynin | Phase 1 | NADPH oxidase inhibitor | - Acetovanillone has been used in trials studying the treatment of bronchial asthma and chronic obstructive pulmonary disease | DrugBank |
|  |  |  | - Apocynin blocked CCL2 production during retinal inflammation | [12] |
|  |  |  | - Apocynin attenuated periorbital mechanical allodynia in relapsing-remitting EAE mouse model | [13] |
| Mocetinostat | Phase 2 | HDAC inhibitor | - Mocetinostat has been used in trials studying the treatment of lymphoma, urothelial carcinoma, relapsed and refractory, myelodysplastic syndrome, and metastatic leiomyosarcoma, among others | DrugBank |
| PAC-1 | Phase 1 | Caspase activator | - PAC-1 has been used in trials studying the treatment of lymphoma, melanoma, solid tumors, breast cancer, and thoracic cancers, among others | DrugBank |
|  |  |  | - B-PAC-1 treatment induced chronic lymphocytic leukemia lymphocyte death which was higher than that in normal peripheral blood mononuclear cells or B cells | [14] |
| GSK-1070916 | Phase 1 | Aurora kinase inhibitor |  |  |
| Piribedil | Launched | Dopamine receptor agonist | - Piribedil has been used with or without levodopa in the treatment of Parkinson's disease | DrugBank |
| Felodipine | Launched | Calcium channel blocker | - Felodipine has been used to treat hypertension | DrugBank |
| PFI-1 | Preclinical | Bromodomain inhibitor | - PFI-1 suppressed plasmablast-mediated plasma cell differentiation in healthy human CD19 B cells and reduced IgG and IgM secretion in costimulation-induced human B cells | [15] |
| Duloxetine | Launched | Norepinephrine reuptake inhibitor  Serotonin-norepinephrine reuptake inhibitor | - Duloxetine has been used to treat generalized anxiety disorder, neuropathic pain, osteoarthritis, and stress incontinence | DrugBank |

Abbreviations: MALT1, mucosa-associated lymphoid tissue lymphoma translocation gene; EAE, Experimental autoimmune encephalomyelitis; DC, Dendritic cell; Th, T helper cell; Treg, Regulatory T cells; RPE, retinal pigment epithelium; EAU, Experimental autoimmune uveitis; EIU, endotoxin-induced uveitis; GABA, γ-aminobutyric acid; NADPH, Nicotinamide Adenine Dinucleotide Phosphate; CCL2, chemokine C-C motif ligand 2; HDAC, histone deacetylase.

| **Table 2 in** **Additional file 12. Results of drug prediction for SCL.** | | | | |
| --- | --- | --- | --- | --- |
| Drugs | Status | Mechanism of action | Relationship with immunity and inflammation | Reference |
| Paricalcitol | Launched | Vitamin D receptor agonist | - Paricalcitol has been used to treat hyperparathyroidism associated with stage 3 or greater chronic kidney disease | DrugBank |
|  |  |  | - Paricalcitol diminished EAE mouse model, as well as macrophages and T cells activation through blocking NF-κB activation | [16] |
| Ampicillin | Launched | Bacterial cell wall synthesis inhibitor | - Ampicillin has been used for the treatment of a variety of infections caused by gram-positive and gram-negative bacteria as well as some anaerobes | DrugBank |
| Enzastaurin | Phase 3 | PKC inhibitor | - Enzastaurin has been used for the treatment of relapsed glioblastoma multiforme | DrugBank |
|  |  |  | - Enzastaurin ameliorated inflammation and clinical symptoms in EAE mouse model, and dose-dependently suppressed T-cell proliferation and cytokine production | [17] |
|  |  |  | - Enzastaurin monotherapy reduced the number of HLA-DR-expressing lymphocytes, suggesting an immunomodulatory effect | [18] |
| Hexachlorophene | Launched | Potassium channel activator | - Hexachlorophene has been used as a surgical scrub and skin cleanser | DrugBank |
| Rilpivirine | Launched | Non-nucleoside reverse transcriptase inhibitor | - Rilpivirine has been used in combination with other antiretrovirals to specifically treat human immunodeficiency virus type 1 | DrugBank |
| ABT-737 | Phase 1/Phase 2 | BCL inhibitor | - ABT-737 was an inhibitor of members of the Bcl‑2 family of apoptosis regulators | DrugBank |
|  |  |  | - ABT-737 reduced disease severity in tissue-specific and systemic animal models of autoimmunity | [19] |
| Dexamethasone-acetate | Launched | Glucocorticoid receptor agonist  Corticosteroid agonist  Cytochrome P450 inhibitor | - Dexamethasone acetate has been used for the treatment of inflammatory respiratory, allergic, autoimmune, and other conditions | DrugBank |
|  |  |  | - Dexamethasone acetate has been an important type of corticoid used in the treatment of some uveitis | [20] |
| Bropirimine | Phase 3 | Interferon inducer | - Bropirimine has been an oral immunostimulant that causes the body to produce interferon and other substances | PubChem |
|  |  |  | - Bropirimine inhibited progression of severe EAE mouse model | [21] |
| Lopinavir | Launched | HIV protease inhibitor | - Lopinavir has been used in combination with ritonavir to treat human immunodeficiency virus infection | DrugBank |
| Everolimus | Launched | MTOR inhibitor | - Everolimus has been used to treat various types of malignancies | DrugBank |
|  |  |  | - Uveitis inactivity was achieved with the addition of everolimus in patients with chronic and cyclosporine A-refractive uveitis | [22] |
|  |  |  | - Topical administration of an everolimus formulation improved EAU in both eyes | [23] |
| Piketoprofen | Launched | Cyclooxygenase inhibitor | - Piketoprofen were non-steroidal anti-inflammatory drugs, often used as topical anti-inflammatory agent | [24] |
| GDC-0941 /Pictilisib | Phase 2 | PI3K inhibitor | - Pictilisib has been used in trials studying the treatment of solid cancers, breast cancer, advanced solid tumors, metastatic breast cancer, and non-Hodgkin's lymphoma, among others | DrugBank |
| OSI-930 | Phase 1 | KIT inhibitor  VEGFR inhibitor | - OSI-930 has been designed to target both cancer cell proliferation and angiogenesis in selected tumors | DrugBank |
| Dehydroepiandrosterone | Launched | Protein synthesis stimulant | - Prasterone has been used for the treatment of moderate to severe dyspareunia associated with menopausal vulvar and vaginal atrophy | DrugBank |
| Ribavirin | Launched | Antiviral | - Ribavirin has been used to treat some forms of Hepatitis C | DrugBank |
| Levonorgestrel | Launched | Estrogen receptor agonist  Glucocorticoid receptor antagonist  Progesterone receptor agonist  Progesterone receptor antagonist | - Levonorgestrel has been used in contraception and hormone therapy | DrugBank |
| PF-573228 | Preclinical | Focal adhesion kinase inhibitor | - PF-573228 reduced chemoattractant-induced migration of B-2 and marginal zone B cells | [25] |
|  |  |  | - PF-573228 impaired T-cell conjugation with antigen-presenting cells | [26] |
| Atovaquone | Launched | Mitochondrial inhibitor | - Atovaquone has been used for the prevention and treatment of pneumocystis jirovecii pneumonia and for the prevention and treatment of plasmodium falciparum malaria | DrugBank |
| Fenigam | Launched | GABA receptor agonist | - Phenibut has been used as an anxiolytic, cognitive enhancer, and alcohol withdrawal treatment | [27] |
| GP-2a | Investigated | Cannabinoid receptor agonist | - Activation of cannabinoid receptors has anti-inflammatory effects | [28] |

Abbreviations: EAE, Experimental autoimmune encephalomyelitis; HLA-DR, human leukocyte antigen DR; PKC, Protein kinase C; Bcl2, B cell lymphoma; EAU, Experimental autoimmune uveitis; HIV, human immunodeficiency virus; MTOR, Mammalian Target of Rapamycin; VEGFR, Vascular endothelial growth factor receptor; GABA, γ-aminobutyric acid.

| **Table 3 in** **Additional file 12.. Results of drug prediction for VKH.** | | | | |
| --- | --- | --- | --- | --- |
| Drugs | Status | Mechanism of action | Relationship with immunity and inflammation | Reference |
| Clonazepam | Launched | GABA receptor agonist | - Clonazepam has been used to treat panic disorders, severe anxiety, and seizures | DrugBank |
| Birinapant | Phase 2 | XIAP inhibitor | - Birinapant has been investigated for the treatment of myelodysplastic syndrome and chronic myelomonocytic leukemia | DrugBank |
| Brivudine | Launched | DNA inhibitor  DNA polymerase inhibitor | - Brivudine has been used to treat herpes zoster | DrugBank |
| L-citrulline | Launched | Nitric oxide stimulant | - Citrulline has been used for nutritional supplementation, also for treating dietary shortage or imbalance | DrugBank |
| Voriconazole | Launched | Cytochrome P450 inhibitor | - Voriconazole has been used to treat fungal infections | DrugBank |
| BRD-K33211335/ Dextromethorph  an | Launched | Glutamate receptor antagonist  Sigma receptor agonist | - Dextromethorphan has been used to treat cases of dry cough | DrugBank |
|  |  |  | - Low dose dextromethorphan attenuated moderate EAE by inhibiting NOX2 and reducing peripheral immune cells infiltration in the spinal cord | [29] |
|  |  |  | - Dextromethorphan exhibited anti-inflammatory and immunomodulatory effects in a murine model of collagen-induced arthritis and in human rheumatoid arthritis | [30] |
| Gossypol | Phase 2 | BCL inhibitor  MCL1 inhibitor | - Gossypol has been used in trials studying the treatment of Non-small cell lung cancer | DrugBank |
|  |  |  | - Gossypol treatment alleviated inflammation, improved the outcomes of CIA mice, impaired the assembly of nuclear receptor coactivator 3-p300-NF-κB complex and decreased the expression of proinflammatory cytokine genes | [31] |
|  |  |  | - Gossypol ameliorated the IL-1β-induced apoptosis and inflammation in chondrocytes by suppressing the activation of TLR4/MyD88/NF-κB pathway via down-regulating connexin43 | [32] |
|  |  |  | - Gossypol showed anti-inflammatory effects on human lymphocytic Jurkat cells via regulation of MAPK signaling and cell cycle | [33] |
| Bivalirudin | Launched | Thrombin inhibitor | - Bivalirudin has been used to treat heparin-induced thrombocytopenia | DrugBank |
| Edaravone | Launched | Nootropic agent | - Edaravone has been used to delay the progression of amyotrophic lateral sclerosis | DrugBank |
|  |  |  | - Edaravone protected against acute autoimmune myocarditis in rats by the radical scavenging action | [34] |
|  |  |  | - Edaravone inhibited CIA possibly through suppression of NF-κB | [35] |
|  |  |  | - Edaravone treatment ameliorated EAE murine model by reducing the infiltration of lymphocytes and the expression of iNOS | [36] |
| Idoxuridin | Launched | DNA synthesis inhibitor | - Idoxuridine has been used for the treatment of viral eye infections, including herpes simplex keratitis | DrugBank |
| Belinostat | Launched | HDAC inhibitor | - Belinostat has been used for the treatment of patients with relapsed or refractory peripheral T-cell lymphoma | DrugBank |
|  |  |  | - Belinostat ameliorated EAE mouse model by inhibiting TLR2/MyD88 and HDAC3/NF-κB p65-mediated neuroinflammation | [37] |
|  |  |  | - Belinostat suppressed DC maturation and regulated immune tolerance of DC | [38] |
| AZD-9291/ Osimertinib | Launched | EGFR inhibitor | - Osimertinib has been used in the treatment of certain types of non-small cell lung carcinoma | DrugBank |
| Cytochalasin D | Launched | Tubulin inhibitor | - Cytochalasin D inhibited ox-LDL phagocytosis, improved autophagic status, and reduced NLRP3 activation and the apoptotic response | [39] |
| VU-0418946-1/ ML228 | Preclinical | Hypoxia inducible factor activator | - ML228 could effectively activate hypoxia-inducible factor and its downstream target VEGF in vitro | [40] |
| Erlotinib | Launched | EGFR inhibitor | - Erlotinib has been used to treat certain small cell lung cancers or advanced metastatic pancreatic cancers | DrugBank |
|  |  |  | - Erlotinib ameliorated CIA mouse model by inhibiting the proliferation and cytokine production of human synovial fibroblasts in vitro | [41] |
|  |  |  | - Erlotinib prevented sclerodermatous graft-versus-host disease in a mouse model by decreasing the production of activated/memory CD4+ T cells and the reduction in T-cell infiltration along with a decrease in IFN-γ and IL-13 production and autoimmune B-cell activation | [42] |
| KIN001-244 |  | PDK1 Inhibitor | - KIN001-244 was investigated as an anti-malarial agent | [43] |
| PD-0325901/ Mirdametinib | Phase 2 | MEK inhibitor | - PD-0325901 has been used in trials studying the treatment and basic science of melanoma, solid tumors, advanced cancer, and breast neoplasms, among others | DrugBank |
|  |  |  | - Mirdametinib treatment inhibited MEK resulting in further suppression of IL-6 and TNF-α levels in vitro | [44] |
| SGX-523 | Phase 1 | HGF receptor inhibitor | - SGX-523 has been investigated for treatment in solid tumors and unspecified cancer/tumors | DrugBank |
| Vorinostat | Launched | HDAC inhibitor | - Vorinostat has been used for the treatment of cutaneous manifestations in patients with progressive, persistent, or recurrent cutaneous T- cell lymphoma following prior systemic therapies | DrugBank |
|  |  |  | - Vorinostat modulated the imbalance of T cell subsets, suppressed macrophage activity, and ameliorated EAU | [45] |
|  |  |  | - Vorinostat attenuated CIA development by inhibition of the Th17 population and maintenance of the Treg population through NR1D1 inhibition | [46] |
|  |  |  | - Vorinostat treatment impaired primary antibody responses in mice, but spared circulating memory B cells | [47] |
|  |  |  | - Vorinostat suppressed DCs and DCs-mediated Th1 and Th17 cell functions and ameliorated EAE | [48] |
| Selumetinib | Phase 3 | MEK inhibitor | - Selumetinib has been used in pediatric patients to treat neurofibromatosis type 1 accompanied by symptomatic, inoperable plexiform neurofibromas | DrugBank |
|  |  |  | - Selumetinib inhibited CD4 T cell activation and T effector cell differentiation | [49] |
|  |  |  | - Selumetinib inhibited N-Formyl peptide receptors-mediated ROS production in fibroblasts derived from systemic sclerosis patients | [50] |

Abbreviations: GABA, γ-aminobutyric acid; XIAP, Recombinant X-linked inhibitor of apoptosis protein; NOX2, Nicotinamide adenine dinucleotide phosphate oxidase 2; EAE, Experimental autoimmune encephalomyelitis; CIA, collagen-induced arthritis; BCL, B cell lymphoma; MCL1, Induced myeloid leukemia cell differentiation protein; iNOS, Inducible nitric oxide synthase; HDAC, histone deacetylase; DC, Dendritic cell; EGFR, Epidermal growth factor receptor; ox-LDL, oxidized low-density lipoprotein; IFN-γ, Interferon γ; IL-13, Interleukin 13; VEGF, Vascular endothelial growth factor; PDK1, Pyruvate dehydrogenase kinase isozyme 1; HGF, Hepatocyte Growth Factor; Th, T helper cell; Treg, Regulatory cells; NR1D1, Nuclear receptor subfamily 1 group D member 1; ROS, reactive oxygen species.

**References in** **supplementary material**

[1] Parreño, M., et al., *Novel triiodophenol derivatives induce caspase-independent mitochondrial cell death in leukemia cells inhibited by Myc.* Mol Cancer Ther, 2006. **5**(5): p. 1166-75.<https://doi.org/10.1158/1535-7163.mct-05-0257>

[2] Mc Guire, C., et al., *Pharmacological inhibition of MALT1 protease activity protects mice in a mouse model of multiple sclerosis.* J Neuroinflammation, 2014. **11**: p. 124.<https://doi.org/10.1186/1742-2094-11-124>

[3] Dumont, C., et al., *A MALT1 inhibitor suppresses human myeloid DC, effector T-cell and B-cell responses and retains Th1/regulatory T-cell homeostasis.* PLoS One, 2020. **15**(9): p. e0222548.<https://doi.org/10.1371/journal.pone.0222548>

[4] Wang, X.C., et al., *Suppression of NF-kappaB-dependent proinflammatory gene expression in human RPE cells by a proteasome inhibitor.* Invest Ophthalmol Vis Sci, 1999. **40**(2): p. 477-86

[5] Zhang, W., et al., *Interplay of Autophagy Inducer Rapamycin and Proteasome Inhibitor MG132 in Reduction of Foam Cell Formation and Inflammatory Cytokine Expression.* Cell Transplant, 2018. **27**(8): p. 1235-1248.<https://doi.org/10.1177/0963689718786229>

[6] Ohkusu-Tsukada, K., D. Ito, and K. Takahashi, *The Role of Proteasome Inhibitor MG132 in 2,4-Dinitrofluorobenzene-Induced Atopic Dermatitis in NC/Nga Mice.* Int Arch Allergy Immunol, 2018. **176**(2): p. 91-100.<https://doi.org/10.1159/000488155>

[7] Hsu, S.M., et al., *Proteasome inhibitor bortezomib suppresses nuclear factor-kappa B activation and ameliorates eye inflammation in experimental autoimmune uveitis.* Mediators Inflamm, 2015. **2015**: p. 847373.<https://doi.org/10.1155/2015/847373>

[8] Chen, F.T., et al., *Anti-inflammatory effect of the proteasome inhibitor bortezomib on endotoxin-induced uveitis in rats.* Invest Ophthalmol Vis Sci, 2012. **53**(7): p. 3682-94.<https://doi.org/10.1167/iovs.12-9505>

[9] Zhang, F., et al., *Puerarin exhibits antiinflammatory properties in gunpowder smog-induced acute lung injury in rats via regulation of the renin-angiotensin system and the NFκB signaling pathway.* Exp Ther Med, 2021. **22**(2): p. 809.<https://doi.org/10.3892/etm.2021.10241>

[10] Komarowska, I., et al., *Hepatocyte Growth Factor Receptor c-Met Instructs T Cell Cardiotropism and Promotes T Cell Migration to the Heart via Autocrine Chemokine Release.* Immunity, 2015. **42**(6): p. 1087-99.<https://doi.org/10.1016/j.immuni.2015.05.014>

[11] Accornero, P., et al., *An in vivo model of Met-driven lymphoma as a tool to explore the therapeutic potential of Met inhibitors.* Clin Cancer Res, 2008. **14**(7): p. 2220-6.<https://doi.org/10.1158/1078-0432.ccr-07-2064>

[12] Zhang, W., et al., *NAD(P)H oxidase-dependent regulation of CCL2 production during retinal inflammation.* Invest Ophthalmol Vis Sci, 2009. **50**(6): p. 3033-40.<https://doi.org/10.1167/iovs.08-2676>

[13] Dalenogare, D.P., et al., *Transient receptor potential ankyrin 1 mediates headache-related cephalic allodynia in a mouse model of relapsing-remitting multiple sclerosis.* Pain, 2022. **163**(7): p. 1346-1355.<https://doi.org/10.1097/j.pain.0000000000002520>

[14] Patel, V., et al., *Expression of executioner procaspases and their activation by a procaspase-activating compound in chronic lymphocytic leukemia cells.* Blood, 2015. **125**(7): p. 1126-36.<https://doi.org/10.1182/blood-2014-01-546796>

[15] Zeng, S., et al., *The suppression of Brd4 inhibits peripheral plasma cell differentiation and exhibits therapeutic potential for systemic lupus erythematosus.* Int Immunopharmacol, 2022. **103**: p. 108498.<https://doi.org/10.1016/j.intimp.2021.108498>

[16] Zhang, D., L. Qiao, and T. Fu, *Paricalcitol improves experimental autoimmune encephalomyelitis (EAE) by suppressing inflammation via NF-κB signaling.* Biomed Pharmacother, 2020. **125**: p. 109528.<https://doi.org/10.1016/j.biopha.2019.109528>

[17] Lanz, T.V., et al., *Protein kinase Cβ as a therapeutic target stabilizing blood-brain barrier disruption in experimental autoimmune encephalomyelitis.* Proc Natl Acad Sci U S A, 2013. **110**(36): p. 14735-40.<https://doi.org/10.1073/pnas.1302569110>

[18] Smetak, M., et al., *Reduction of HLA-DR+ lymphocytes after treatment with enzastaurin in patients with metastatic thyroid cancer.* Chemotherapy, 2008. **54**(4): p. 268-73.<https://doi.org/10.1159/000149717>

[19] Bardwell, P.D., et al., *The Bcl-2 family antagonist ABT-737 significantly inhibits multiple animal models of autoimmunity.* J Immunol, 2009. **182**(12): p. 7482-9.<https://doi.org/10.4049/jimmunol.0802813>

[20] da Silva, G.R., et al., *Effect of the macromolecular architecture of biodegradable polyurethanes on the controlled delivery of ocular drugs.* J Mater Sci Mater Med, 2009. **20**(2): p. 481-7.<https://doi.org/10.1007/s10856-008-3607-y>

[21] Vroegop, S.M., et al., *Pharmacology of the biological response modifier bropirimine (PNU-54461) on experimental autoimmune encephalomyelitis (EAE) in mice.* Int J Immunopharmacol, 1999. **21**(6): p. 391-409.<https://doi.org/10.1016/s0192-0561(99)00019-3>

[22] Heiligenhaus, A., et al., *Everolimus for the treatment of uveitis refractory to cyclosporine A: a pilot study.* Graefes Arch Clin Exp Ophthalmol, 2013. **251**(1): p. 143-52.<https://doi.org/10.1007/s00417-012-2163-9>

[23] Kasper, M., et al., *Novel everolimus-loaded nanocarriers for topical treatment of murine experimental autoimmune uveoretinitis (EAU).* Exp Eye Res, 2018. **168**: p. 49-56.<https://doi.org/10.1016/j.exer.2018.01.003>

[24] López-Abad, R., et al., *Topical dexketoprofen as a cause of photocontact dermatitis.* J Investig Allergol Clin Immunol, 2004. **14**(3): p. 247-9

[25] Tse, K.W., et al., *Small molecule inhibitors of the Pyk2 and FAK kinases modulate chemoattractant-induced migration, adhesion and Akt activation in follicular and marginal zone B cells.* Cell Immunol, 2012. **275**(1-2): p. 47-54.<https://doi.org/10.1016/j.cellimm.2012.03.002>

[26] Wiemer, A.J., et al., *The focal adhesion kinase inhibitor PF-562,271 impairs primary CD4+ T cell activation.* Biochem Pharmacol, 2013. **86**(6): p. 770-81.<https://doi.org/10.1016/j.bcp.2013.07.024>

[27] Peterkin, A.F., R. Abraham, and M.T.H. Harris, *A Case of Phenibut Directed Detoxification Leading to Toxicity During the COVID-19 Pandemic.* J Addict Med, 2022. **16**(5): p. 602-605.<https://doi.org/10.1097/adm.0000000000000966>

[28] Turcotte, C., et al., *The CB(2) receptor and its role as a regulator of inflammation.* Cell Mol Life Sci, 2016. **73**(23): p. 4449-4470.<https://doi.org/10.1007/s00018-016-2300-4>

[29] Chechneva, O.V., et al., *Low dose dextromethorphan attenuates moderate experimental autoimmune encephalomyelitis by inhibiting NOX2 and reducing peripheral immune cells infiltration in the spinal cord.* Neurobiol Dis, 2011. **44**(1): p. 63-72.<https://doi.org/10.1016/j.nbd.2011.06.004>

[30] Chen, D.Y., et al., *Dextromethorphan Exhibits Anti-inflammatory and Immunomodulatory Effects in a Murine Model of Collagen-Induced Arthritis and in Human Rheumatoid Arthritis.* Sci Rep, 2017. **7**(1): p. 11353.<https://doi.org/10.1038/s41598-017-11378-8>

[31] Sun, X., et al., *Nuclear receptor coactivator 3 transactivates proinflammatory cytokines in collagen-induced arthritis.* Cytokine, 2023. **161**: p. 156074.<https://doi.org/10.1016/j.cyto.2022.156074>

[32] Li, S., et al., *Gossypol ameliorates the IL-1β-induced apoptosis and inflammation in chondrocytes by suppressing the activation of TLR4/MyD88/NF-κB pathway via downregulating CX43.* Tissue Cell, 2021. **73**: p. 101621.<https://doi.org/10.1016/j.tice.2021.101621>

[33] Chen, C.W., et al., *Anti-inflammatory Effects of Gossypol on Human Lymphocytic Jurkat Cells via Regulation of MAPK Signaling and Cell Cycle.* Inflammation, 2018. **41**(6): p. 2265-2274.<https://doi.org/10.1007/s10753-018-0868-6>

[34] Nimata, M., et al., *MCI-186 (edaravone), a novel free radical scavenger, protects against acute autoimmune myocarditis in rats.* Am J Physiol Heart Circ Physiol, 2005. **289**(6): p. H2514-8.<https://doi.org/10.1152/ajpheart.00661.2005>

[35] Arii, K., et al., *Edaravone inhibits collagen-induced arthritis possibly through suppression of nuclear factor-kappa B.* Mol Immunol, 2008. **45**(2): p. 463-9.<https://doi.org/10.1016/j.molimm.2007.05.020>

[36] Moriya, M., et al., *Edaravone, a free radical scavenger, ameliorates experimental autoimmune encephalomyelitis.* Neurosci Lett, 2008. **440**(3): p. 323-6.<https://doi.org/10.1016/j.neulet.2008.05.110>

[37] Shen, Y., et al., *The histone deacetylase inhibitor belinostat ameliorates experimental autoimmune encephalomyelitis in mice by inhibiting TLR2/MyD88 and HDAC3/ NF-κB p65-mediated neuroinflammation.* Pharmacol Res, 2022. **176**: p. 105969.<https://doi.org/10.1016/j.phrs.2021.105969>

[38] Jia, W.H., et al., *[Study on the immune functions of dendritic cells regulated by histone deacetylase inhibitor Belinostat].* Zhonghua Xue Ye Xue Za Zhi, 2018. **39**(1): p. 41-46.<https://doi.org/10.3760/cma.j.issn.0253-2727.2018.01.009>

[39] Zheng, L., et al., *Intervention time decides the status of autophagy, NLRP3 activity and apoptosis in macrophages induced by ox-LDL.* Lipids Health Dis, 2022. **21**(1): p. 107.<https://doi.org/10.1186/s12944-022-01714-x>

[40] Theriault, J.R., et al., *Discovery of a new molecular probe ML228: an activator of the hypoxia inducible factor (HIF) pathway.* Bioorg Med Chem Lett, 2012. **22**(1): p. 76-81.<https://doi.org/10.1016/j.bmcl.2011.11.077>

[41] Swanson, C.D., et al., *Inhibition of epidermal growth factor receptor tyrosine kinase ameliorates collagen-induced arthritis.* J Immunol, 2012. **188**(7): p. 3513-21.<https://doi.org/10.4049/jimmunol.1102693>

[42] Morin, F., et al., *Inhibition of EGFR Tyrosine Kinase by Erlotinib Prevents Sclerodermatous Graft-Versus-Host Disease in a Mouse Model.* J Invest Dermatol, 2015. **135**(10): p. 2385-2393.<https://doi.org/10.1038/jid.2015.174>

[43] KalantarMotamedi, Y., et al., *A systematic and prospectively validated approach for identifying synergistic drug combinations against malaria.* Malar J, 2018. **17**(1): p. 160.<https://doi.org/10.1186/s12936-018-2294-5>

[44] Chen, Q., et al., *Inhibition Ras/MEK/ERK pathway: An important mechanism of Baihu Jia Guizhi Decoction ameliorated rheumatoid arthritis.* J Ethnopharmacol, 2022. **304**: p. 116072.<https://doi.org/10.1016/j.jep.2022.116072>

[45] Fang, S., et al., *Vorinostat Modulates the Imbalance of T Cell Subsets, Suppresses Macrophage Activity, and Ameliorates Experimental Autoimmune Uveoretinitis.* Neuromolecular Med, 2016. **18**(1): p. 134-45.<https://doi.org/10.1007/s12017-016-8383-0>

[46] Kim, D.S., et al., *Suberoylanilide Hydroxamic Acid Attenuates Autoimmune Arthritis by Suppressing Th17 Cells through NR1D1 Inhibition.* Mediators Inflamm, 2019. **2019**: p. 5648987.<https://doi.org/10.1155/2019/5648987>

[47] Waibel, M., et al., *Manipulation of B-cell responses with histone deacetylase inhibitors.* Nat Commun, 2015. **6**: p. 6838.<https://doi.org/10.1038/ncomms7838>

[48] Ge, Z., et al., *Vorinostat, a histone deacetylase inhibitor, suppresses dendritic cell function and ameliorates experimental autoimmune encephalomyelitis.* Exp Neurol, 2013. **241**: p. 56-66.<https://doi.org/10.1016/j.expneurol.2012.12.006>

[49] Zheng, R., et al., *Spermine alleviates experimental autoimmune encephalomyelitis via regulating T cell activation and differentiation.* Int Immunopharmacol, 2022. **107**: p. 108702.<https://doi.org/10.1016/j.intimp.2022.108702>

[50] Napolitano, F., et al., *N-Formyl Peptide Receptors Induce Radical Oxygen Production in Fibroblasts Derived From Systemic Sclerosis by Interacting With a Cleaved Form of Urokinase Receptor.* Front Immunol, 2018. **9**: p. 574.<https://doi.org/10.3389/fimmu.2018.00574>
